# Disruption of tubulin-alpha4a polyglutamylation prevents aggregation of hyper-phosphorylated tau and microglia activation in mice

**DOI:** 10.1038/s41467-022-31776-5

**Published:** 2022-07-20

**Authors:** Torben Johann Hausrat, Philipp C. Janiesch, Petra Breiden, David Lutz, Sabine Hoffmeister-Ullerich, Irm Hermans-Borgmeyer, Antonio Virgilio Failla, Matthias Kneussel

**Affiliations:** 1 Department of Molecular Neurogenetics, Center for Molecular Neurobiology, ZMNH, University Medical Center Hamburg-Eppendorf, Falkenried 94, 20251 Hamburg, Germany; 2 Department of Structural Neurobiology, ZMNH, University Medical Center Hamburg-Eppendorf, Falkenried 94, 20251 Hamburg, Germany; 3 Bioanalytics Facility, Center for Molecular Neurobiology, ZMNH, University Medical Center Hamburg-Eppendorf, Falkenried 94, 20251 Hamburg, Germany; 4 Transgenic Animal Unit, Center for Molecular Neurobiology, ZMNH, University Medical Center Hamburg-Eppendorf, Falkenried 94, 20251 Hamburg, Germany; 5 Microscopy Imaging Facility, University Medical Center Hamburg-Eppendorf, Martinistraße 52, 20251 Hamburg, Germany; 6 Present Address: Department of Neuroanatomy and Molecular Brain Research, Ruhr University Bochum, 44801 Bochum, Germany

**Keywords:** Alzheimer's disease, Cellular neuroscience

## Abstract

Dissociation of hyper-phosphorylated Tau from neuronal microtubules and its pathological aggregates, are hallmarks in the etiology of tauopathies. The Tau-microtubule interface is subject to polyglutamylation, a reversible posttranslational modification, increasing negative charge at tubulin C-terminal tails. Here, we asked whether tubulin polyglutamylation may contribute to Tau pathology in vivo. Since polyglutamylases modify various proteins other than tubulin, we generated a knock-in mouse carrying gene mutations to abolish Tuba4a polyglutamylation in a substrate-specific manner. We found that Tuba4a lacking C-terminal polyglutamylation prevents the binding of Tau and GSK3 kinase to neuronal microtubules, thereby strongly reducing phospho-Tau levels. Notably, crossbreeding of the Tuba4a knock-in mouse with the hTau tauopathy model, expressing a human *Tau* transgene, reversed hyper-phosphorylation and oligomerization of Tau and normalized microglia activation in brain. Our data highlight tubulin polyglutamylation as a potential therapeutic strategy in fighting tauopathies.

## Abstract (web summary)

Pathologic oligomerization of hyper-phosphorylated Tau is a hallmark of tauopathies. Here the authors show that the loss of tubulin a4 polyglutamylation reverses tau hyperphosphorylation, oligomerization and microglia activation in a tauopathy mouse.

## Introduction

A growing number of neurodegenerative diseases summarized as tauopathies are linked to the dysfunction of Tau, a microtubule-associated protein (MAP) also known as MAP-T^1–4^. They include Alzheimer´s disease (AD), corticobasal degeneration, or frontotemporal dementia, which are characterized by improper accumulation of hyper-phosphorylated Tau, known as neurofibrillary tangles (NFTs) or Pick bodies. Tau accumulations are accompanied by microglia activation, which potentially induces gliosis and neurodegeneration in brain^5,6^. While microglial activation follows the aggregation of phosphorylated Tau in tauopathy models^7^, proinflammatory cytokines, released by activated microglia, contribute to Tau hyper-phosphorylation and pathology^8^. Glycogen synthase kinase-3 (GSK3), also associating with microtubules, is a major kinase catalyzing Tau phosphorylation^9–11^. Upon hyper-phosphorylation, axonal Tau detaches from microtubules, however, the physiological mechanisms that regulate their interaction are still incompletely understood^5^.

The microtubule cytoskeleton represents a complex and dynamic structure in eukaryotes, regulating a diversity of cellular functions. Microtubules maintain the structure of cells, form spindles in mitosis and meiosis and provide the tracks for intracellular transport^12,13^. In neurons, representing polarized and excitable cells, microtubules contribute to the formation and maintenance of axons and dendrites, regulate the polarized delivery and removal of cargoes over long distances and transiently invade into actin-rich dendritic spines in a neuronal activity-dependent manner^14–17^. At the molecular level, microtubules are composed of alpha- and beta-tubulin isotypes that heterodimerize in a 1:1 stoichiometry^18^. Tubulin dimers polymerize into protofilaments in a GTP-dependent manner, with typically 13 protofilaments assembling into a hollow tube structure, representing the microtubule. So far, eight genes encoding alpha-tubulins and eight genes encoding beta-tubulins have been described, which are differentially expressed during development and in different brain regions to form functional microtubules^12,19,20^.

Microtubule-associated proteins (MAPs) regulate microtubule organization by different mechanisms and/or modulate the binding and function of interacting proteins^21–25^. For instance, Tau is a microtubule bundler that stimulates growth and inhibits shrinkage of microtubules^25^ through regulation of their labile domain^24^.

Microtubules are subject to posttranslational modifications (PTMs) such as phosphorylation, acetylation, de-tyrosination, polyglycination or polyglutamylation^26–31^. Polyglutamylation at tubulin C-termini differentially regulates the interaction of microtubules with MAPs in vitro^32^, modifies the function of severing enzymes or motor proteins^33–35^ and potentially alters dendritic and axonal transport^33,36^. Polyglutamylation is catalyzed in a reversible manner through a variety of TTLL-type glutamylases (for tubulin tyrosine ligase-like) and CCP-type de-glutamylases (for cytoplasmic carboxypeptidase), respectively^27^. However, not all TTLL members are glutamylases. In fact, TTLL3, TTLL8, and TTLL10 represent polyglycylases whereas the specific function of TTLL12 is presently unknown^37^. Several attempts to investigate the functional role of tubulin polyglutamylation have been limited by the fact that TTLLs modify various protein substrates other than tubulin. This includes the nucleosome assembly proteins NAP1 and NAP2, the nucleophosmin B23, the microtubule +TIP protein EB1, the myosin light chain kinase (MLCK), and the retinitis pigmentosa GTPase regulator (RPGR)^38–42^. Consequently, loss-of-function approaches with respect to TTLL enzymes lead to overlapping effects that are unspecific.

In order to unravel the functional role of tubulin polyglutamylation in the mouse brain, we developed a substrate-specific genetic approach. Generating a knock-in mouse that carries C-terminal *Tuba4a* point mutations (Tuba4aΔpolyGlu), we interfered with polyglutamylation specifically at Tuba4a-containing microtubules in vivo. Neuronal Tuba4a isotypes contain the longest polyglutamyl side chains across the tubulin family^43^. The gene expression pattern of *Tuba4a* significantly increases at adult stages, leading to the most abundant levels of polyglutamylation, as compared to other tubulins^19,43^. Notably, Tuba4a is the only tubulin that lacks a C-terminal tyrosine residue and is therefore independent of another reversible tubulin PTM, known as de-tyrosination.

Our data show that the binding of Tau and GSK3 kinase to microtubules containing mutant Tuba4a is severely decreased, with Tau remaining in a hypo-phosphorylated state. This finding was unexpected, since pathologic Tau aggregating in tauopathies is strongly hyper-phosphorylated. Strikingly, crossbreeding of *Tuba4a* knock-in mutants with a tauopathy model, expressing a human *Tau* transgene, diminishes Tau pathology and microglia activation. Our data provide mechanistic insights into the Tau-microtubule interface and highlight isotype-specific polyglutamylation of tubulin as a potential molecular target to develop treatment strategies against tauopathies.

## Results

### Generation of knock-in mice characterized by a specific loss of Tuba4a C-terminal polyglutamylation

**Fig. 1:**
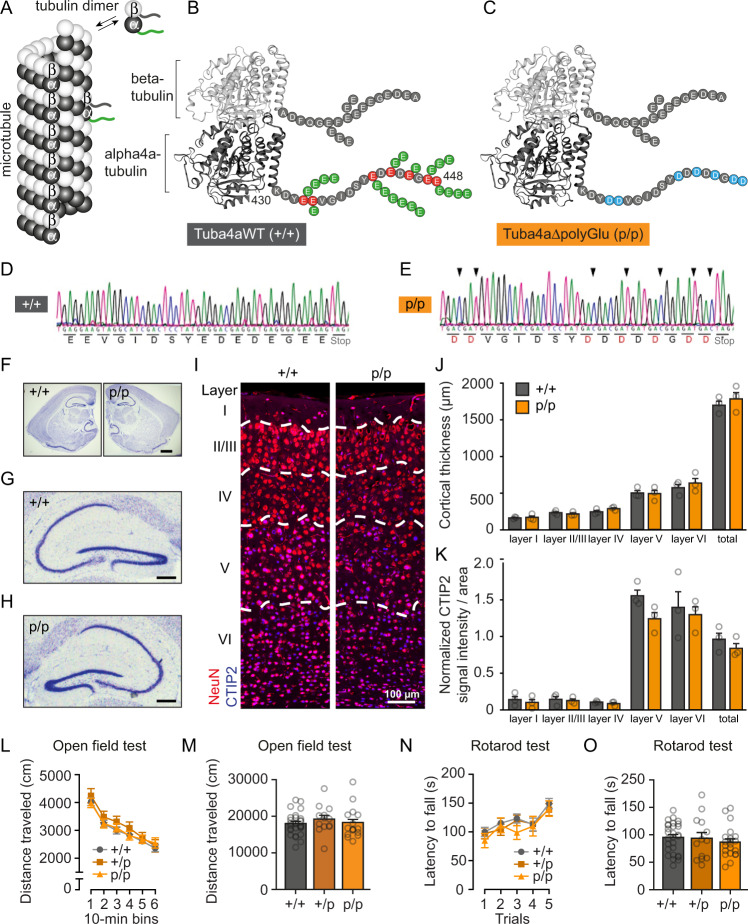
Development of a knock-in mouse, carrying point mutations to preclude polyglutamylation of Tuba4a. **A** Microtubules assemble from heterodimers of alpha- and beta-tubulin. C-terminal tubulin tails are subject to different posttranslational modifications (PTMs). **B**, **C** To preclude Tuba4a polyglutamylation at glutamate residues (green circles), 7 final glutamate residues (red circles) are genetically substituted by aspartate (blue circles). PTMs of other tubulin isotypes remain unaffected. **D**, **E** DNA sequencing. Amino acid substitutions are indicated (red letters). **F** Representative Nissl staining of coronal brain sections from adult mice, derived from three independent experiments. Scale bar, 1 mm. **G**, **H** Magnification of hippocampal regions shown in (**F**). Scale bar, 250 µm. **I**–**K** Immunohistochemical analysis of cortical layering based on NeuN and CTIP2 in cortical brain sections derived from 1-year-old Tuba4aΔpolyGlu (+/+) and (p/p) mice. Scale bar, 100 µm. Quantification of (**J**) individual cortical layer thickness and (K) CTIP2 signal intensities normalized to the area analyzed, as indicated. *n* = 3 mice per genotype. **L**, **M** Open field test to study locomotor activity in 6-month-old Tuba4aΔpolyGlu (+/+), (+/p) and (p/p) mice. No differences regarding **L** the distance traveled over 10-min bins and (**M**) total distance traveled over 60 min in the open field between genotypes. Main effect for genotype: *F*_2,56_ = 0.501; *p* = 0.608; Main effect for bins × genotype: *F*_10,280_ = 0.408; *p* = 0.942; *n*(+/+) = 28, n(+/p) = 13, *n*(p/p) = 21. **N**, **O** Accelerating rotarod test. Latency to fall off the rod measures balance and motor learning. No differences regarding (**N**) the latency to fall over 5 trials and (**O**) mean latency to fall over 5 trials on the rotarod between genotypes. Main effect for genotype: *F*_2,58_ = 0.498; p = 0.610; Main effect for trials × genotype: *F*_8,220_ = 0.409; *p* = 0.914; *n*(+/+) = 28, *n*(+/p) = 13, *n*(p/p) = 21. Two-sided unpaired Student’s *t*-test (**J**, **K**) and ANOVA (**M**, **O**) and two-way repeated measure ANOVA (**L**, **N**) were used to assess statistical significance. Data represent mean ± SEM. Source data, including exact *p*-values, are provided as a Source Data file.

In order to investigate tubulin polyglutamylation in a substrate-specific manner, we eliminated the respective target sites at the C-terminal tail of a unique tubulin substrate. To this end, we generated gene-targeted knock-in mice, carrying a group mutation (glutamate (E) to aspartate (D)) in the mouse *Tuba4a* gene, encoding alpha4a-tubulin (Tuba4a) (Fig. 1A–C, green tails and Supplementary Fig. 1A, B). Gene-targeted mice were crossbred with flp-Deleter and Cre-deleter mice to generate constitutive knock-in mutants (Supplementary Fig. 1C), hereafter referred to as Tuba4aΔpolyGlu. Southern blotting at the ES cell stage (Supplementary Fig. 1D–E) and a long-range PCR over the 3’integration site (Supplementary Figs. 1C, F) confirmed the correct insertion of the targeting vector. This result was verified using genomic DNA from knock-in mouse tissue (Supplementary Fig. 1G, H). Sequencing of the respective gene segment in *Tuba4a* revealed the presence of aspartate (D) residues in the knock-in (p/p for present/present) allele (Fig. 1E), as compared to the wild-type (+/+) allele (Fig. 1D), whereas PCR genotyping identified wild-type (+/+), heterozygous (+/p) and homozygous (p/p) genotypes (Supplementary Fig. 1I). Mutant mice carrying the *Tuba4a* group mutation developed normally. They produced litters of similar size on average (Supplementary Fig. 1J) with an expected Mendelian ratio of genotypes (Supplementary Fig. 1K, L). They further showed no gross abnormalities in the brain and hippocampal morphology, as assessed by Nissl staining (Fig. 1F–H). Likewise, their cortical layering assessed by NeuN and CTIP2 immunostaining was normal (Fig. 1I–K). To test locomotor activity and exploratory behavior, mutant mice (+/p and p/p) and wild-type (+/+) littermates were assessed in the open field test, revealing no differences across genotypes (Fig. 1L, M). They further showed a similar motor coordination and motor learning in the rotarod test (Fig. 1N, O), indicative of normal motor cortex development and function.

**Fig. 2:**
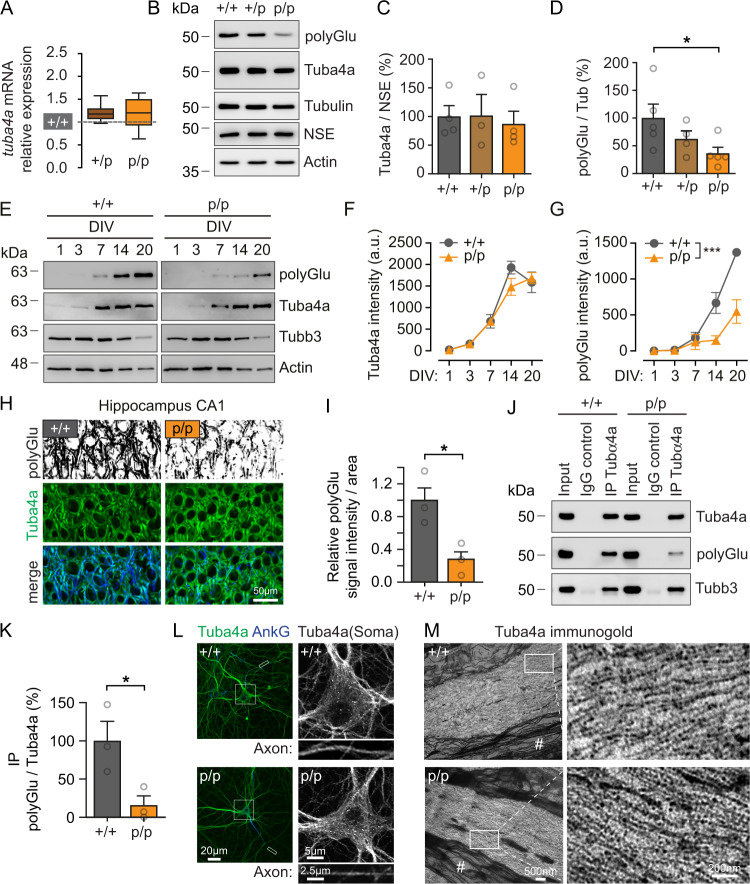
The *Tuba4a* mutation reduces overall tubulin polyglutamylation levels but is still incorporated into microtubules. **A** Relative *Tuba4a* mRNA expression levels. (+/+) set to 1 (gray dotted line) in adult mice. Median (center), interquartile range (bounds of boxes), and minima and maxima (whiskers) are indicated. *n* = 11 samples per genotype. Two-sided alternative hypothesis (H1) testing was used to assess statistical significance. **B** Representative western blot analysis depicting tubulin polyglutamylation (polyGlu), Tuba4a, total alpha-tubulin, NSE and Actin in adult Tuba4aΔpolyGlu (+/+), (+/p) and (p/p) mice. Please note that the remaining levels of polyglutamylation are due to tubulins other than Tuba4a. **C**, **D** Quantification of Tuba4a normalized to NSE (tubulin-independent loading control) (**C**) and polyglutamylated tubulin normalized to total alpha-tubulin (**D**) signal intensities shown in (**B**). (+/+) set to 100%. **C** *n* = 4(+/+ and p/p), 3(+/p) and **D** *n* = 5(+/+ and p/p), 4(+/p) experiments per genotype. **E** Developmental expression of polyGlu, Tuba4a, Tubb3, and Actin in hippocampal neurons at different DIV, as indicated. **F**, **G** Quantification of Tuba4a (**F**) and polyglutamylated tubulin (**G**) signal intensities shown in (**E**) over time. Two-way ANOVA (DIV × genotype) with *p* = 0.2 (Tuba4a) and *p* = 0.0004 (polyGlu), *n* = 3 independent cultures per genotype per time point. **H** Immunohistochemical analysis of Tuba4a (green) and polyGlu (greyscale or blue) in CA1 region of hippocampal brain sections derived from 12-month-old Tuba4aΔpolyGlu (+/+) and (p/p) mice. Scale bar, 50 µm. **I** Quantification of polyGlu normalized to the area analyzed, shown in (**H**). +/+ set to 1; *n* = 3 mice per genotype. **J** Representative coIP of Tuba4a and Tubb3 from hippocampal lysates derived from adult mice. In addition, polyglutamylated tubulin levels were analyzed. **K** Quantification of signal intensities of precipitated polyGlu normalized to signal intensities of precipitated Tuba4a, shown in (**J**), *n* = 3 experiments. **L** Coimmunostaining of Tuba4a (green) and AnkyrinG (blue; axonal marker) using DIV14 neurons. *n* = 3 experiments. Scale bars, 20 µm (overview), 5 µm (soma), 2.5 µm (axon). **M** High-resolution immunogold electron microscopy (EM) of Tuba4a in the medulla oblongata (rich in parallel axons) from adult Tuba4aΔpolyGlu (+/+) and (p/p) mice. Micrographs on the left show magnifications of the white rectangles. The presence of myelin sheets (#) identifies axons. Note, the pearl necklace-like distribution of gold particles at microtubules. Scale bars, 500 nm (overview), 200 nm (magnification). *n* = 3 mice per genotype. Two-sided unpaired Student’s *t*-test (**A**, **C**, **D**, **I**, **K**) and ANOVA (**F**, **G**) were used to assess statistical significance. *p < 0.05, ***p < 0.001. Data represent mean ± SEM, if not stated otherwise. Source data, including exact p-values, are provided as a Source Data file.

The analysis of *Tuba4a* mRNA (Fig. 2A) and protein levels (Fig. 2B, C) in the hippocampus revealed no significant differences between genotypes, indicating that the group mutation does not alter *Tuba4a* gene expression. As expected, detection of polyglutamylated tubulin (polyGlu)^44,45^ displayed a clear reduction in polyGlu levels (Fig. 2B, D (p/p)), suggesting that Tuba4a, as characterized by comparatively long poly-Glu side chains^43^, is a major substrate of polyglutamylating enzymes. At the same time, other tubulin PTMs remained unchanged in Tuba4a mutants (Supplementary Fig. 2A–E). In neuronal cultures, detection of Tuba4a and polyGlu tubulin over in vitro development (DIV1-20), revealed a gradual increase in polyglutamylated tubulin (polyGlu, (+/+) Fig. 2E–G) that was also significantly reduced in neurons from homozygous Tuba4a mutants (polyGlu, (p/p), Fig. 2E–G). In line with this, reduced tubulin polyglutamylation in Tuba4a mutants was also detectable by immunostaining of hippocampal slices derived from adult mice (Fig. 2H, I), while Tuba4a signal intensities remained unchanged (Supplementary Fig. 2F). Furthermore, using an immunoprecipitation experiment to enrich Tuba4a from hippocampal brain lysate, we confirmed a strong reduction of polyglutamylation specifically for the precipitated tubulin isotypes (Fig. 2J, middle (p/p), 2 K). Notably, Tubb3 co-immunoprecipitated with Tuba4a using brain lysate from mutant (p/p) mice (Fig. 2J, lower), indicating that C-terminal Tuba4a polyglutamylation is not a prerequisite to dimerize with beta-tubulin. Using immunostaining, we detected Tuba4a in neuronal dendrites and in Ankyrin-G (AnkG) -positive axons (Fig. 2L). As expected, Tuba4a signals decorated microtubule filaments in neuronal somata of both genotypes (Fig. 2L), confirming the incorporation of wild-type and mutant Tuba4a into the microtubule polymer. This could be corroborated by Tuba4a immunogold electron microscopy, which labeled tubulin-alpha4a along individual microtubules of both genotypes at ultrastructural resolution (Fig. 2M). Together, we conclude that the Tuba4aΔpolyGlu mouse lacks tubulin polyglutamylation at Tuba4a C-termini (Figs. 1E and 2B, D, E, G–K), while mutant Tuba4a still dimerizes with beta-tubulin (Fig. 2J) and integrates into microtubules (Fig. 2L, M).

### Loss of Tuba4a C-terminal polyglutamylation alters microtubule growth but not KIF5C-mediated transport

**Fig. 3:**
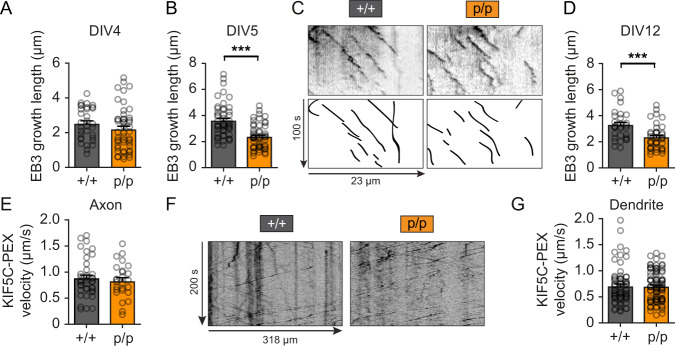
Loss of Tuba4a C-terminal polyglutamylation alters microtubule growth without affecting kinesin motor protein processivity. **A**–**D** Live imaging of EB3-GFP in hippocampal neurons derived from Tuba4aΔpolyGlu (+/+) and (p/p) mice. Quantification of EB3-GFP growth length 24 h after transfection at DIV4 (**A**), DIV5 (**B**) and DIV12 (**D**). Representative kymographs acquired from dendrites at DIV5 are shown in (**C**). Upper panels: GFP signal, lower panels: tracked EB3-GFP. Analyzed comets from 19 to 24 cells from three independent cultures per genotype per DIV: DIV4, *n*(+/+) = 32, *n*(p/p) = 48; DIV5, *n*(+/+) = 49, *n*(p/p) = 53; DIV12, n(+/+) = 30, *n*(p/p) = 40. **E**–**G** Quantification of KIF5C motility (velocity in µm/s) in axons (**E**) and dendrites (**G**) at DIV13 using KIF5C without a cargo-binding domain fused to tdTomato and pex26 (peroxisome binding domain) expressed for 24 h in hippocampal neurons derived from Tuba4aΔpolyGlu (+/+) and (p/p) mice. Representative kymographs of KIF5C in dendrites are shown in (**F**). Analyzed tracks from 17 to 23 cells from three independent cultures per genotype: Axon, *n*(+/+) = 40, *n*(p/p) = 26; Dendrite, *n*(+/+) = 68, *n*(p/p) = 89. Two-sided Mann–Whitney test was used to assess statistical significance. ***p < 0.001. Data represent mean ± SEM. Source data, including exact *p*-values, are provided as a Source Data file.

To ask whether polyglutamylation at the Tuba4a C-terminus regulates microtubule growth and/or kinesin-mediated transport, we performed neuronal live-cell imaging with cultured hippocampal neurons. The use of a fluorescent microtubule +TIP protein EB3 (EB3-GFP) revealed a significant reduction in the growth length and growth duration of microtubules, using neurons from Tuba4aΔpolyGlu (p/p) mice at stage DIV5 and DIV12 (Fig. 3B–D, Supplementary Fig. 3D–I). Consistent with Tuba4a being hardly expressed at earlier stages (compare with Fig. 2E, F), this effect was not detectable at DIV4 (Fig. 3A, Supplementary Fig. 3A–C). These data, therefore, suggest that the regulation of microtubule dynamics is sensitive to Tuba4a polyglutamylation. In contrast, the Tuba4a C-terminal mutations had no effect on KIF5-mediated transport velocities along axonal or dendritic microtubules in mutant (p/p) neurons, following expression of a KIF5C-PEX construct that connects the motor to peroxisomes (Fig. 3E–G).

### Analysis of MAP- and Tau-microtubule binding in the absence of Tuba4a C-terminal polyglutamylation

**Fig. 4:**
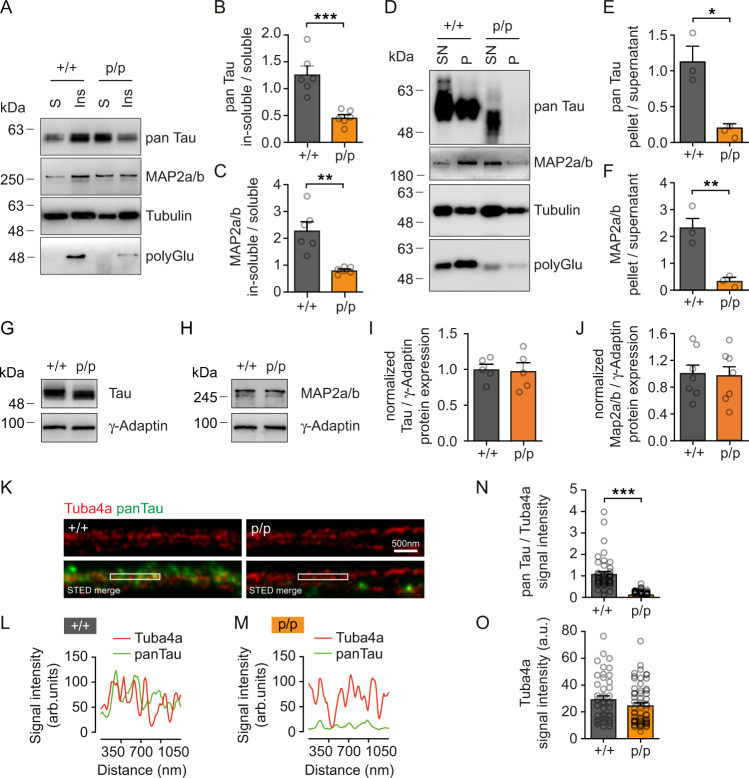
Loss of Tuba4a C-terminal polyglutamylation significantly decreases Tau binding to microtubules. **A**–**C** Soluble-tubulin extraction assay using DIV15 neurons derived from Tuba4aΔpolyGlu (+/+) and (p/p) mice. **A** Western blot. Soluble fraction (S): un-polymerized tubulin and dissociated MAPs. Insoluble fraction (Ins): polymerized tubulin (microtubules) and associated MAPs. Fractions were probed for pan Tau, MAP2a/b, total alpha-tubulin, and polyglutamylated tubulin (polyGlu). Quantification of pan Tau (**B**) and MAP2a/b (C) in-soluble/soluble levels. Ratios smaller than 1 indicate higher protein abundance in the soluble fraction. *n* = 6 independent cultures per genotype. **D**–**F** Microtubule pelleting assay after repolymerization of hippocampal tubulin derived from adult mice. **D** Western blot. Supernatant (SN): un-polymerized tubulin and dissociated MAPs. Pellet (P): polymerized tubulin (microtubules) and associated MAPs. Fractions were probed for pan Tau, MAP2a/b, total alpha-tubulin and polyGlu. Quantification of pan Tau (**E**) and MAP2a/b (**F**) pellet/supernatant levels. Ratios smaller than 1 indicate higher protein abundance in the supernatant fraction. *n* = 3 mice per genotype. **G**–**J** Representative western blot analysis depicting Tau (**G**) and MAP2a/b (**H**) normalized to γ-Adaptin protein expression levels in the hippocampus. Respective quantifications are shown in (**I**, **J**). (+/+) set to 1. *n* = 5–7 experiments. **K** Representative super-resolution STED images of microtubules in axonal regions from DIV14 hippocampal neurons, derived from three independent experiments. Tuba4a (red), pan Tau (green). **L**, **M** Line scans: relative intensities of signals along 1 μm of microtubule length (boxed regions in (**K**)). Scale bar, 500 nm. Arbitrary units (arb. units). **N** Mean pan Tau signal intensities normalized to Tuba4a. *n* = 45(+/+), 60(p/p) axonal regions. **O** Total Tuba4a signal intensities. *n* = 45(+/+), 60(p/p) axonal regions. Two-sided unpaired Student´s t-test (**B**, **C**, **E**, **F**, **I**, **J**) and Mann–Whitney test (**N**, **O**) were used to assess statistical significance. *p < 0.05, **p < 0.01, ***p < 0.001. Data represent mean ± SEM. Source data, including exact *p*-values, are provided as a Source Data file.

Previous biochemical in vitro studies had suggested that polyglutamylation acts like a rheostat regulating the affinity between tubulin and Tau or MAP2^32,46^. To test this hypothesis in the Tuba4aΔpolyGlu mouse, we initially performed a soluble tubulin extraction assay using hippocampal neurons. Tau, MAP2a/b, and polyglutamylated tubulin (polyGlu) were prominent in the wildtype (+/+) in-soluble fraction enriched in polymerized microtubules (Fig. 4A, left). In contrast, neurons from mutant mice (p/p), characterized by a strongly reduced polyGlu signal (Fig. 4A, bottom right, compare with Fig. 2B and J), displayed a >50% reduction in Tau-microtubule (Fig. 4A top right and B) and MAP2-microtubule (Fig. 4A, C) co-occurrence in the in-soluble fraction. This result was even more striking in a microtubule pelleting experiment, following repolymerization of microtubules. Whereas Tau was highly abundant in pellets from wild-type (+/+) fractions, containing strong polyGlu signals (Fig. 4D left and E), Tau binding to microtubules was hardly detectable in the pellet of fractions derived from Tuba4a mutant (p/p) mice (Fig. 4D right and E), as characterized by weak polyglutamylation (Fig. 4D, bottom, right) but equal tubulin levels (Supplementary Fig. 4A). Likewise, MAP2a/b binding to microtubules was severely reduced under these conditions (Fig. 4D, F), whereas MAP1a binding to microtubules remained unchanged (Supplementary Fig. 4B, C), indicating a differential effect of Tuba4a C-terminal polyglutamylation on MAP/microtubule interaction. These effects were not due to changes in gene expression, since the relative expression levels of Tau, MAP2a/b and MAP1a remained equal in both genotypes (Fig. 4G–J and Supplementary Fig. 4D–G).

Immunocytochemical analysis revealed equal Tau levels in axons of wild-type and mutant neurons (Supplementary Fig. 4H, I), suggesting that the axonal localization of Tau is independent of Tuba4a polyglutamylation. In contrast, super-resolution STED microscopy confirmed a loss of colocalization between endogenous Tuba4a and endogenous Tau at microtubule filaments. The signal intensities for the colocalization of both proteins were significantly reduced in neurons from Tuba4a mutant (p/p) mice, as compared to wild-type (+/+) controls (Fig. 4K (yellow signal), Fig. 4L–N, Supplementary Fig. 4J). In contrast, the signal intensities of Tuba4a (red channel) remained equal, confirming that Tuba4a itself was not downregulated (Fig. 4O). We, therefore, conclude that efficient Tau binding to microtubules requires a polyglutamylated Tuba4a C-terminus.

### Analysis of Tau phosphorylation in the absence of Tuba4a C-terminal polyglutamylation

**Fig. 5:**
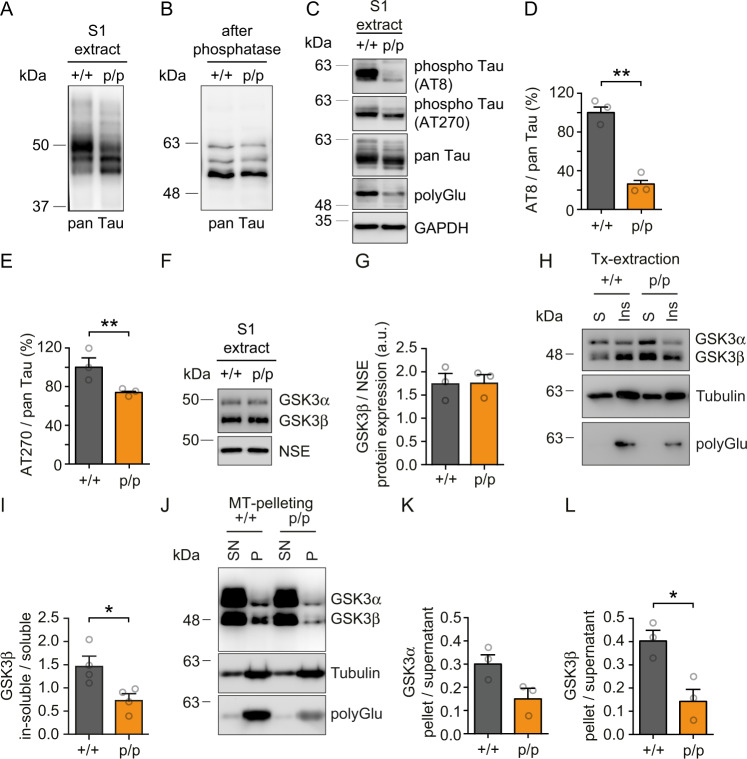
Loss of Tuba4a C-terminal polyglutamylation significantly decreases phosphorylation of mouse Tau and binding of GSK3β to microtubules. **A** Representative western blot analysis of pan Tau in hippocampal lysates (S1 extract) from adult Tuba4aΔpolyGlu (+/+) and (p/p) mice. **B** Representative western blot analysis of pan Tau in hippocampal lysates (S1 extract) after phosphatase treatment from adult mice. **C** Western blot analysis of Tau phosphorylation in hippocampal lysates (S1 extract) from adult mice. AT8 and AT270: phosphorylated Tau-specific antibodies. pan-Tau: total-Tau. polyGlu: polyglutamylated tubulin. GAPDH: loading control. The representative western blots shown in (**A**–**C**) are derived from three independent experiments. **D**–**E** Quantification of relative AT8 (**D**) and AT270 (**E**) signal intensities normalized to total Tau. (+/+) set to 100%. *n* = 3 experiments per genotype. **F** Western blot analysis of GSK3α/β in hippocampal lysates (S1 extract) from adult mice. NSE: loading controls. **G** Quantification of GSK3β signal intensities shown in (**F**). *n* = 3 mice per genotype. **H**, **I** Soluble-tubulin extraction assay using DIV15 neurons derived from Tuba4aΔpolyGlu (+/+) and (p/p) mice. (H) Western blot. Soluble fraction (S): un-polymerized tubulin and dissociated MAPs. Insoluble fraction (Ins): polymerized tubulin (microtubules) and associated MAPs. Fractions were probed for GSK3α/β and total alpha-tubulin and polyGlu tubulin. **I** Quantification of GSK3β in-soluble/soluble levels. Ratios smaller than 1 indicate higher protein abundance in soluble fractions. *n* = 4 independent cultures per genotype. **J**–**L** Microtubule pelleting assay following re-polymerization of hippocampal tubulin from adult mice. **J** Western blot. The supernatant (SN): un-polymerized tubulin and dissociated MAPs. Pellet (P) polymerized tubulin (microtubules) and associated MAPs. Fractions were probed for GSK3α/β, polyGlu and total alpha-tubulin. **K** Quantification of GSK3α pellet/supernatant levels. **L** Quantification of GSK3β pellet/supernatant levels. Ratios smaller than 1 indicate higher protein abundance in supernatant fractions. *n* = 3 mice per genotype. Two-sided unpaired Student’s *t*-test was used to asses statistical significance. *p < 0.05, **p < 0.01. Data represent mean ± SEM. Source data, including exact *p*-values, are provided as a Source Data file.

The inability of Tau to efficiently bind and colocalize with non-polyglutamylated Tuba4a prompted us to analyze the Tau phosphorylation status, since Tau hyper-phosphorylation and microtubule-detachment are hallmarks in Alzheimer´s disease (AD) and other tauopathies^2,47,48^. Interestingly, western blotting with a pan Tau antibody, using hippocampal extracts derived from wild-type (+/+) or Tuba4aΔpolyGlu mutant (p/p) mice, led to the detection of different molecular weights of individual Tau variants with or without Tuba4a C-terminal polyglutamylation, respectively (Fig. 5A). This result was due to changes in the phosphorylation status of Tau, since phosphatase treatment, resulting in the de-phosphorylation of Tau, led to an equal pattern in both genotypes (Fig. 5B). Notably, application of the phospho-specific Tau antibodies AT8 and AT270, known to detect individual phospho-residues within the protein (Supplementary Table 1)^49,50^, revealed significantly reduced Tau phosphorylation levels in Tuba4a mutant (p/p) fractions at both epitopes (Fig. 5C–E), whereas Tau expression per se remained unaltered (Fig. 5C, pan Tau detection and Fig. 4G, I and Supplementary Fig. 4F). Together, these data suggest that a non-polyglutamylated Tuba4a C-terminus not only prevents Tau binding (Fig. 4), but also interferes with Tau phosphorylation.

Therefore, we analyzed two major Tau kinases GSK3α and β in wild-type (+/+) and Tuba4aΔpolyGlu mutant (p/p) mice. Protein expression levels of GSK3α and β were found to be equal in hippocampal lysates derived from either genotype (Fig. 5F, G, Supplementary Fig. 5A). On the other hand, attachment of GSK3α and β at microtubules was significantly reduced in a soluble-tubulin extraction assay derived from mutant (p/p) neurons (Fig. 5H, I, Supplementary Fig. 5B), an effect that was not due to changes in tubulin levels (Supplementary Fig. 5C, D). This result was confirmed using an independent microtubule pelleting assay from the hippocampal lysate, following repolymerization of microtubules. In this experiment, we also detected significantly less GSK3β associated with microtubules, as compared to a tubulin loading control (Fig. 5J–L).

Together, our data reveal that the *Tuba4a* mutation in this study (Fig. 1A–E), which interferes with C-terminal tubulin polyglutamylation in mice (Fig. 2), significantly alters (i) Tau binding to microtubules (Fig. 4), (ii) Tau phosphorylation (Fig. 5A–E) and (iii) GSK3-microtubule interactions (Fig. 5H–L). They highlight the critical importance of Tuba4a polyglutamylation in regulating the Tau-microtubule interface in vivo.

### Loss of Tuba4a C-terminal polyglutamylation leads to reduced phosphorylation levels of human Tau

To investigate whether the Tuba4a C-terminus may be a potential target sequence in developing treatment strategies against tauopathies, we aimed to study human Tau and its tendency to form hyper-phosphorylated oligomers, in Tuba4aΔpolyGlu (p/p) mice.

**Fig. 6:**
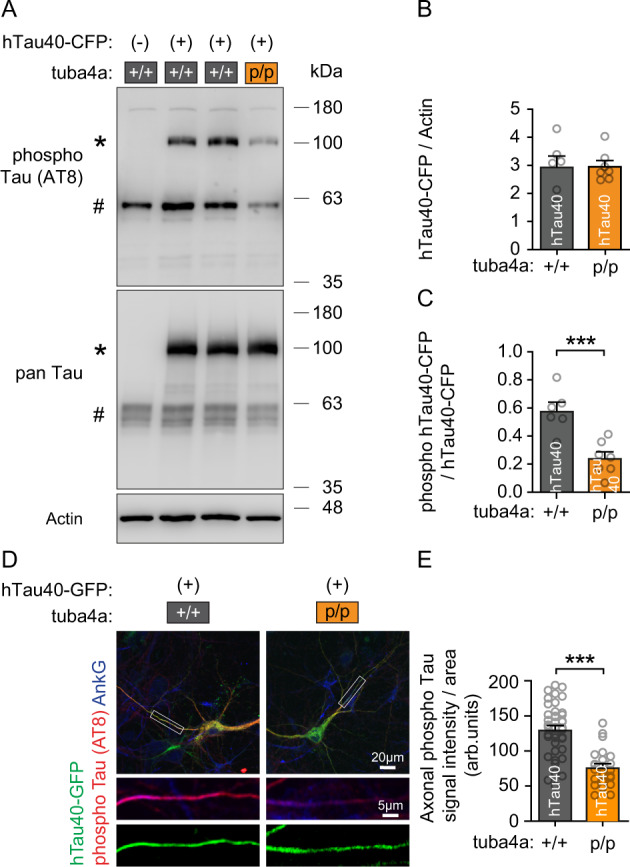
Hyper-phosphorylation of human Tau is normalized upon the loss of Tuba4a C-terminal polyglutamylation. **A** Western blot of mock- or adeno virus-transduced CFP-tagged human Tau (hTau40-CFP) using DIV19 neurons from Tuba4aΔpolyGlu (+/+) and (p/p) mice after 2 days of expression. AT8: phosphorylated Tau-specific antibody. pan-Tau: total Tau. Loading control: Actin. Note, endogenous mouse Tau (#) and the human CFP-Tau fusion protein (*) are distinguishable based on their different molecular weights. The representative western blots shown in (**A**) are derived from three independent experiments. **B** hTau40-CFP signal intensities normalized to actin. *n* = 6(+/+), 7(p/p) cultures per genotype. **C** Phospho-hTau40-CFP signal intensities normalized to total Tau, as shown in (**A**). *n* = 6(+/+), 7(p/p) cultures per genotype. **D** Representative immunostaining of phosphorylated Tau (AT8, red), using DIV10 hippocampal neurons derived from Tuba4aΔpolyGlu (+/+) and (p/p) mice, 2 days after transfection of hTau40-GFP constructs (green signals). Scale bars, 20 µm (overview) and 5 µm (magnification). Ankyrin-G (AnkG, blue) was used as an axonal marker. **E** Quantification of phosphorylated Tau signal intensities normalized to the axonal area analyzed, as shown in (**D**). *n* = 41(+/+), 25(p/p) cells per genotype. Arbitrary units (arb. units). A two-sided unpaired Student´s t-test was used to asses statistical significance. ***p < 0.001. Data represent mean ± SEM. Source data, including exact p-values, are provided as a Source Data file.

To this end, we virally expressed CFP-tagged human Tau (hTau40-CFP) in neurons derived from wild-type (+/+) or Tuba4a mutant (p/p) mice. Due to its CFP-tag, human Tau40 displayed a higher molecular weight (*), compared to endogenous mouse Tau (#) (Fig. 6A). Infected wild-type (+/+) and Tuba4a mutant (p/p) neurons expressed the hTau40-CFP fusion protein and mouse Tau at similar levels (Fig. 6A, lower pan-Tau detection, 6B and Supplementary Fig. 6A). In contrast, hTau40 phospho-levels normalized to total hTau40-CFP levels were significantly reduced in Tuba4a mutant (p/p) neurons (Fig. 6A, upper AT8 detection; Fig. 6C), suggesting that the loss of Tuba4a C-terminal polyglutamylation might protect against hyper-phosphorylation of human Tau. Using immunocytochemistry, reduced Tau phospho-levels could also be confirmed in axons of Tuba4a mutant (p/p) neurons expressing human Tau (hTau40-GFP). In wild-type neurons, Tau phosphorylation (red AT8 signals) in the axon was relatively high after hTau40-GFP expression, but significantly reduced in Tuba4a mutant (p/p) neurons under the same conditions (Fig. 6D, E).

### Loss of Tuba4a C-terminal polyglutamylation normalizes Tau hyper-phosphorylation and its abnormal oligomerization in a tauopathy model

**Fig. 7:**
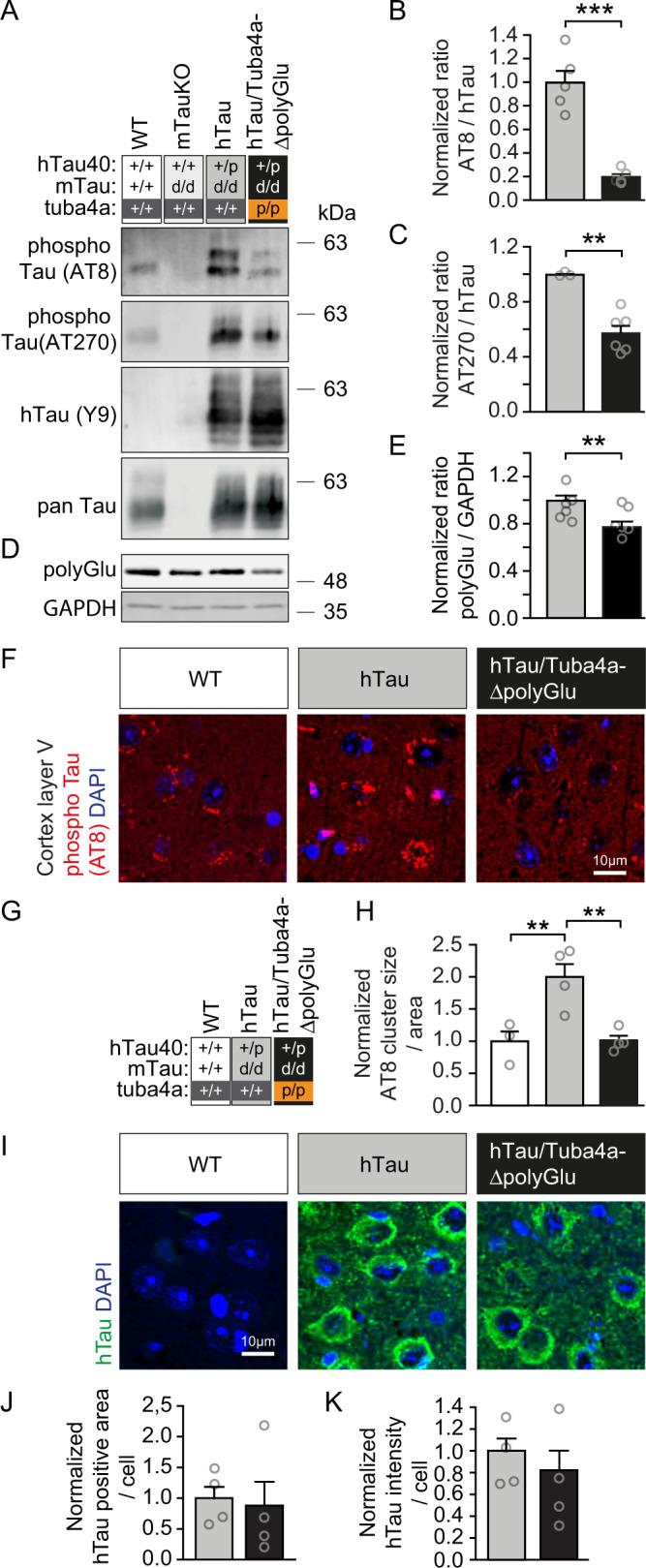
Hyper-phosphorylation of human Tau in a tauopathy mouse model is normalized upon the loss of Tuba4a C-terminal polyglutamylation. **A** Western blot analysis of hTau phosphorylation using hippocampal lysates from 12-month-old mice. Tuba4aΔpolyGlu (+/+) and (p/p) mice were crossed with a tauopathy mouse-model (hTau (+/p); mTau (d/d)). The mouse Tau knockout condition (mTauKO (d/d)) indicates antibody specificity. AT8 and AT270: phosphorylated Tau. Y9: total human Tau. pan Tau: total Tau. **B**, **C** Quantification of phosphorylated Tau signal intensities, probed with AT8 (**B**) or AT270 (**C**), normalized to hTau, as shown in (**A**). hTau mice (dark gray condition) set to 1; (**B**) *n* = 5 experiments, **C** *n* = 3(+/+), 6(p/p) experiments. **D** Western blot analysis depicting polyglutamylated tubulin (polyGlu). GAPDH: loading control. **E** Quantification of polyGlu signal intensities normalized to GAPDH. hTau mice (dark gray condition) set to 1, *n* = 6 mice per genotype. Note, human Tau hyper-phosphorylation is normalized in Tuba4a (p/p) as compared to Tuba4a (+/+) derived hippocampal lysates. **F** Immunohistochemical analysis of subcellular phosphorylated Tau-positive accumulations in layer V of cortical brain sections derived from 12-month-old Tuba4a (+/+) and (p/p) mice crossed with hTau (+/p) mice. Wild-type mice not expressing hTau, served as controls. AT8 (red): phospho-Tau. DAPI (blue): nuclei. Scale bar, 10 µm. **G** Legend indicating group (condition)/genotype assignment. **H** Quantification of AT8-positive accumulations size per area analyzed, shown in (**F**). WT set to 1; *n* = 3(WT), 4(+/+ and p/p) mice per genotype. Note, Tau hyper-phosphorylation is normalized in the genetic background of Tuba4aΔpolyGlu. **I** Immunohistochemical analysis of subcellular Tau aggregates in layer V of cortical brain sections derived from 12-month-old Tuba4a (+/+) and (p/p) mice crossed with hTau (+/p) mice. Wild-type sections not expressing hTau, served as antibody control. hTau (green): total human Tau. DAPI (blue): nuclei. Scale bar, 10 µm. **J**, **K** Quantification of hTau-positive accumulations size per area analyzed (**J**) and signal intensities per area (**K**) analyzed, shown in (**I**). WT set to 1; *n* = 4 mice per genotype. Two-sided unpaired Student’s *t*-test (**B**–**E**, J–**K**) and one-way ANOVA (**H**) were used to asses statistical significance. **p < 0.01, ***p < 0.001. Data represent mean ± SEM. Source data, including exact *p*-values, are provided as a Source Data file.

In a further approach, we crossbred wild-type (+/+) or Tuba4a mutant mice (p/p) with a tauopathy mouse model (hTau), known for its Alzheimer´s disease-like pathology, as characterized by the pathologic oligomerization of hyper-phosphorylated Tau in cell bodies and dendrites. Genetically, hTau mice represent homozygous knockouts for mouse Tau, carrying one allele of a human *Tau40* transgene^51^. As expected, hTau (dark gray) and hTau/Tuba4aΔpolyGlu (black) animals containing an *hTau40* allele (+/p), overexpressed hTau40 in the hippocampus (Fig. 7A, Y9 detection) at equal levels (Supplementary Fig. 7H, I). Similar to Tuba4aΔpolyGlu (p/p) mice (Fig. 2D–I, orange conditions), hTau/Tuba4aΔpolyGlu animals were also characterized by significantly reduced polyglutamylation levels (Fig. 7D, E, black condition), whereas related tubulins or other tubulin PTM levels were equal across genotypes (Supplementary Fig. 7A–G). Strikingly, following crossbreeding of Tuba4aΔpolyGlu (p/p) mice with hTau tauopathy mice, the phosphorylation of Tau (AT8 and AT270) was significantly reduced in the resulting hTau/Tuba4a-ΔpolyGlu condition, (Fig. 7A, black condition and Fig. 7B, C), as compared to the tauopathy hTau condition only (dark gray condition). We, therefore, conclude that the loss of Tuba4a C-terminal polyglutamylation (Tuba4aΔpolyGlu) is sufficient to normalize the hyper-phosphorylation Tau phenotype, characteristic of the hTau tauopathy model^51^, in vivo.

To confirm this result, we stained cortical and hippocampal brain slices using the phosphorylated Tau-specific antibody AT8. According to the literature^51^, we detected a significant increase in cortical cells containing hyper-phosphorylated Tau, using the tauopathy model (hTau), as compared to wild-type (WT) controls. This increase was most prominent in layer V of the cortex (Supplementary Fig. 8A, B, dark gray header). Notably, cells from hTau/Tuba4aΔpolyGlu mice (black header), lacking Tuba4a polyglutamylation, resembled those from wild-type mice (white header), indicating a significant normalization of the pathologic hTau phenotype in vivo (Supplementary Fig. 8A, B). We also analyzed phosphorylated Tau levels at higher magnification within neuronal somata. Cells in the tauopathy model (hTau), were characterized by prominent hyper-phosphorylation in perinuclear regions of their somata (Fig. 7F–H, Supplementary Fig. 9A–C, gray header), which was significantly lower in wild-type cells (white header). Again, this pathological phenotype was normalized to control levels in the absence of Tuba4a polyglutamylation (Fig. 7F–H and Supplementary Fig. 9A–C, hTau/Tuba4aΔpolyGlu mice, black header). However, hTau mice were not yet characterized by changes in hTau expression and aggregation (Fig. 7I–K and Supplementary Fig. 9D–E) and revealed a normal thickness of cortical layers (Supplementary Fig. 10), suggesting that at this stage of analysis neurodegeneration is not yet prominent.

**Fig. 8:**
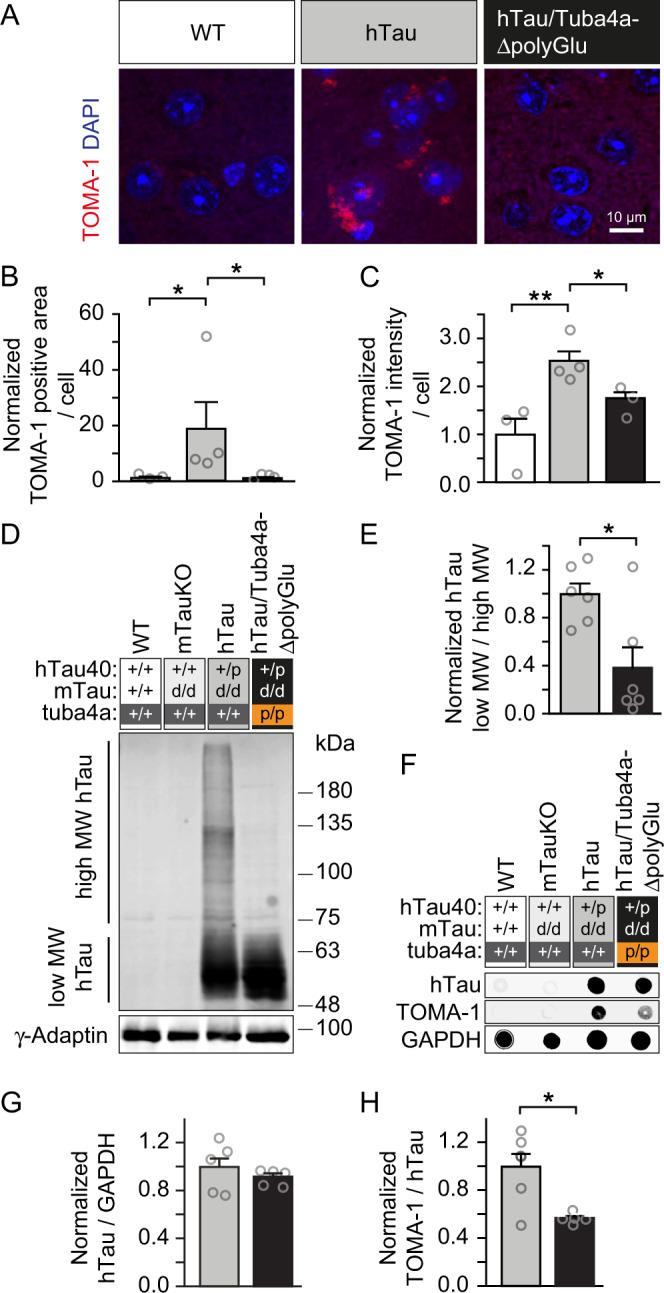
Tau oligomerization in a tauopathy mouse model is normalized upon the loss of Tuba4a C-terminal polyglutamylation. **A** Immunohistochemical analysis of subcellular Tau oligomerization in layer V of cortical brain sections derived from 12-month-old Tuba4a (+/+) and (p/p) mice crossed with hTau (+/p) mice. Wild-type sections not expressing hTau, served as antibody control. TOMA-1 (red): specifically detects oligomerized human Tau. DAPI (blue): nuclei. Scale bar, 10 µm. **B**, **C** Quantification of oligomerized hTau-positive accumulations (**B**) and signal intensities (**C**) normalized to the total area/cell analyzed, shown in (**A**). WT set to 1; *n* = 3(WT), 4(+/+ and p/p) mice per genotype. **D** Western blot analysis depicting hTau, separated using a semi-denaturating-PAGE to preserve protein oligomerization derived from cortical lysates from 12-month-old mice, genotypes are indicated. **E** Quantification of the ration of low and high molecular weight (MW) hTau signal intensities, as shown in (**D**). Note, the abundance of high MW hTau is normalized in the genetic background of Tuba4aΔpolyGlu. hTau mice (dark gray condition) set to 1; *n* = 6 mice per genotype. **F** Dot blot analysis using cortical lysates from 12-month-old mice probed with TOMA-1 (oligomerized Tau), hTau (total human Tau) and GAPDH (loading control), genotypes are indicated. **G** Quantification of hTau signal intensities normalized to GAPDH. hTau mice (dark gray condition) set to 1; *n* = 6(+/+), 5(p/p) mice per genotype. **H** Quantification of TOMA-1 (oligomerized human Tau) signal intensities normalized to hTau (total human Tau). hTau mice (dark gray condition) set to 1; *n* = 6(+/+), 5(p/p) mice per genotype. Two-sided Kruskal Wallis test (**B**, **C**), Mann Whitney test (**E**, **G**) and unpaired Student’s *t*-test (**H**) were used to assess statistical significance. *p < 0.05, **p < 0.01. Data represent mean ± SEM. Source data, including exact *p*-values, are provided as a Source Data file.

We, therefore, asked whether the abnormal oligomerization of human hyper-phosphorylated Tau, often referred to as pre-tangles, a premature state of neurofibrillary tangles (NFTs)^52^, might also be normalized in hTau/Tuba4aΔpolyGlu mice. To this end, we applied TOMA-1 antibodies specific for the detection of oligomeric forms of Tau^53^. Similar to the normalization of hyper-phosphorylated Tau (Fig. 7F–H and Supplementary Fig. 9A–C), hTau/Tuba4aΔpolyGlu animals, lacking Tuba4a polyglutamylation revealed normalization of Tau oligomeric assemblies, as compared to hTau tauopathy mice (Fig. 8A–C). Human Tau oligomerization could further be confirmed in a western blot-based experiment using the hTau antibody. As expected, WT and mTauKO animals did not display any hTau expression (Fig. 8D, white and light gray condition), whereas hTau animals displayed bands at the higher molecular weight (MW) above 75 kDa (Fig. 8D, dark gray condition), indicative of Tau oligomers. Consistent with the TOMA-1 immunostainings (Fig. 8A–C), the intensity of these hTau-specific high MW signals, was significantly decreased in hTau/Tuba4aΔpolyGlu animals lacking Tuba4a polyglutamylation (Fig. 8D, E, black condition). Finally, the use of the TOMA-1 antibody in dot blot assays corroborated Tau oligomers in hTau animals (Fig. 8F–H, dark gray condition) that were significantly reduced in hTau/Tuba4aΔpolyGlu mice (Fig. 8F–H, black condition).

Together, our data point to Tuba4a polyglutamylation as a potential parameter to preclude the hyper-phosphorylation and oligomerization of Tau (Fig. 10).

### Loss of Tuba4a C-terminal polyglutamylation normalizes microglia activation in a tauopathy model

**Fig. 9:**
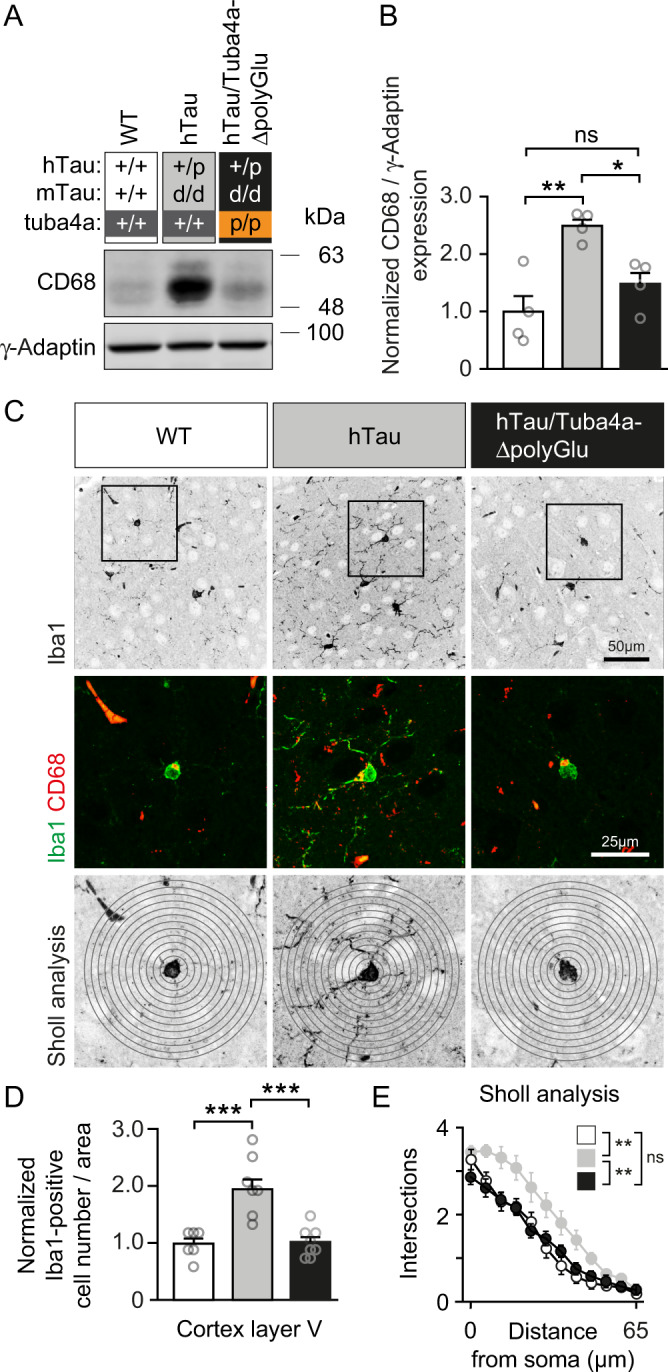
Microglia activation and expansion in a tauopathy mouse model is normalized upon the loss of Tuba4a C-terminal polyglutamylation. **A** Western blot analysis of CD68 (marker for activated microglia) in cortical lysates derived from 12-month-old Tuba4a (WT) and Tuba4aΔpolyGlu mice crossed with a tauopathy mouse model (hTau (+/p)). Wild-type mice not expressing hTau, served as controls. **B** CD68 signal intensities normalized to γ-Adaptin, shown in (**A**). Tuba4a (WT) set to 1; *n* = 4 mice per genotype. **C** Iba1-positive cells (marker for microglia), in cortical brain sections derived from 12-month-old Tuba4a (WT) and Tuba4aΔpolyGlu mice, crossed with hTau (+/p) mice. Wild-type mice not expressing hTau, served as controls. Upper panels: Iba1 (greyscale) within layer V, scale bar, 50 µm. Middle and lower panels: magnification of boxed regions in upper panels; Iba1 (green), CD68 (red); scale bar, 25 µm. Lower panels: Sholl analysis with 6 µm intervals (circles). **D** Normalized cell number of Iba1-positive cells per area analyzed in cortical layer V. *n* = 6(WT), 8(+/+ and p/p) per genotype. **E** Quantification of Sholl analysis: the number of microglia branch intersections that occur from the soma in concentric circles was analyzed. Higher values reflect complex microglia processes, indicating activated microglia. Note that gliosis, as detectable in hTau cortical sections, is significantly reduced in the background of Tuba4aΔpolyGlu, as compared to Tuba4a (WT). *n* = 6(WT), 8(+/+ and p/p) per genotype. Two-sided one-way (**B**, **D**) or two-way (**E**; genotype × distance) ANOVA was used to assess statistical significance. *p < 0.05, **p < 0.01, ***p < 0.001. Data represent mean ± SEM. Source data, including exact *p*-values, are provided as a Source Data file.

Microglia activation contributes to gliosis in response to neuronal damage and is a common pathological feature in tauopathies and AD^54^, accompanied by the aggregation of hyper-phosphorylated Tau^7^. As expected, the hTau tauopathy model displayed strong levels of CD68 (Fig. 9A, B, gray header), a marker for activated microglia and actively phagocytic cells^55^. In contrast, hTau/Tuba4aΔpolyGlu mice, lacking Tuba4a polyglutamylation (black header), displayed significantly less CD68 expression, similar to wild-type controls (white header) (Fig. 9A, B). Coimmunostaining of CD68 with Iba1, a marker highlighting microglia morphology^56^, revealed that activated microglial cells had formed long branching processes in the tauopathy model that were marginally developed in hTau/Tuba4aΔpolyGlu mice (Fig. 9C, green processes). The number of Iba1-positive cells in the cortex (Fig. 9D, Supplementary Fig. 10), as well as the number of branch intersections following a Sholl analysis in cortical layer V (Fig. 9C, E), turned out to be normalized upon the loss of Tuba4a C-terminal polyglutamylation.

**Fig. 10:**
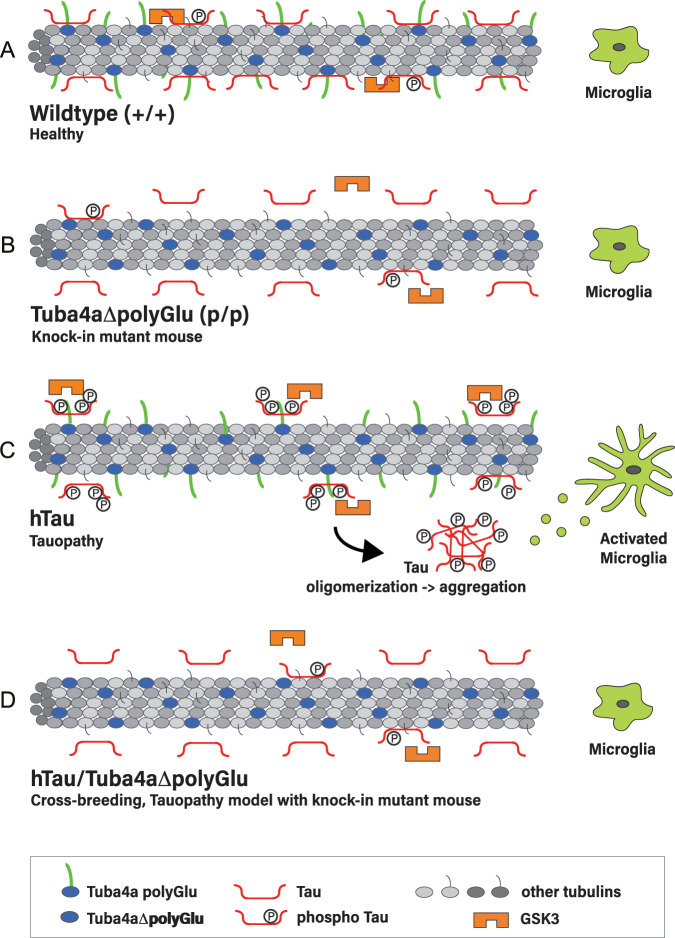
Model. **A** In healthy wild-type neurons, tubulin C-termini are posttranslationally modified through polyglutamylation (tails). Tau binds to microtubules and can be phosphorylated by the kinase GSK3. **B** The knock-in mutant mouse (Tuba4aΔpolyGlu), generated in this study, lacks polyglutamylation (green tails in **A**), specifically at tubulin alpha-4 (Tuba4, blue). Mutant mice are characterized by reduced Tau and GSK3 binding to microtubules. **C** The tauopathy mouse model hTau is characterized by hyper-phosphorylated Tau that detaches from microtubules, oligomerizes and subsequently forms Tau-aggregates. Neurons containing oligomerized Tau are accompanied by microglia activation, leading to gliosis and neurodegeneration (tauopathy). **D** Crossbreeding of hTau and Tuba4aΔpolyGlu mutant mice normalizes the disease phenotype of hTau conditions. Similar to wild-type mice, double mutants contain (i) normal phosphorylated Tau levels, (ii) normal levels of oligomerized Tau and (iii) normal microglia activation patterns. These data suggest that polyglutamylation is a critical parameter in the etiology of tauopathies.

It is therefore plausible to conclude that blocking polyglutamylation at the Tuba4a C-terminus protects neuronal tissue against the consequences of reactive microglia in a tauopathy model (Fig. 10).

## Discussion

In this study, we aimed to investigate how polyglutamylation of tubulin affects microtubules and their downstream functions in neurons and the brain. Since the enzymes that reversibly catalyze polyglutamylation (TTLLs and CCPs) modify multiple substrates, genetic modification or functional inhibition of these proteins leads to a variety of unspecific effects. In the present study, we, therefore, applied a substrate-specific approach, generating a knock-in mouse to study the loss of polyglutamylation at a selected tubulin isotype. We identified C-terminal polyglutamylation of Tuba4a as a critical factor, in regulating the Tau-microtubule interface. Loss of Tuba4a C-terminal polyglutamylation impaired Tau phosphorylation and severely reduced Tau binding to microtubules. Following crossbreeding with a tauopathy mouse model, the *Tuba4a* mutation prevented hyper-phosphorylation and oligomerization of Tau and normalized microglia activation and gliosis within the cortex, a major indicator preceding neurodegeneration. These data point to Tuba4a polyglutamylation as a potential target in fighting tauopathies.

Eight individual isotypes of alpha- and beta-tubulin are described. They are differentially expressed during development and in individual brain regions, mediating unique isotype-specific functions^19^. The unique identity of individual tubulin isotypes is extended through different posttranslational modifications, such as polyglutamylation, acetylation, and/or de-tyrosination^17,27,28^. Our data support and extend this view providing a specific role of Tuba4a C-terminal polyglutamylation in regulating the microtubule-associated protein Tau under pathological conditions. Tuba4a is a major tubulin isotype, highly expressed in the postnatal nervous system^19,57,58^. It is the only tubulin isotype that lacks a tyrosine residue at its very C-terminal end and is therefore independent of de-tyrosination, another tubulin PTM^19,29,30^. Among the different tubulin isotypes expressed in the brain, Tuba4a carries the longest polyglutamyl side chains^43,59^, which is a unique characteristic within the family of tubulins. Since TTLL enzymes are suggested to tolerate amino acid substitutions within tubulin C-termini and seem to modify neighboring glutamate residues instead^60,61^, we substituted seven C-terminal glutamate residues, in this study. This *Tuba4a* group mutation does neither interfere with tubulin dimerization, nor with microtubule incorporation (Fig. 2) and therefore preserves general tubulin functions. Consistent with the fact that TTLL enzymes still polyglutamylate other alpha- and beta-tubulin isotypes^62^, we observe a reduction but not a total loss of tubulin polyglutamylation in Tuba4aΔpolyGlu derived tissues.

Early work suggested that proteolytic removal of the complete C-terminal tubulin tail by subtilisin decreases the mobility of kinesin and dynein motor proteins that move along such microtubules^63^. In vitro studies with engineered chains of glutamate residues confirmed and extended this view, reporting differential effects on motor protein processivity. In fact, long chains of glutamate reduced the processivity of kinesin-1 but not kinesin-2, whereas dynein processivity was increased^34^. Moreover, a cellular study reported that increased tubulin polyglutamylation, induced by synaptic activity, correlated with inhibition of kinesin-1, but not kinesin-3 processivity^33^. These data were supported by reduced mitochondrial transport in a double knockout of the de-glutamylases CCP1/CCP6, which induced tubulin hyper-glutamylation^64^. Although previous cellular and in vivo investigations suggest a functional relevance for the polyglutamylation of “tubulin”, none of them has in fact been substrate specific, taking into account that other subcellular and cytoskeletal regulators, such as the +TIP protein EB1 or the myosin light chain kinase (MLCK), also undergo polyglutamylation by the same family of enzymes^38–42^. Genetic approaches at the substrate level, such as the present study, are therefore required to pinpoint open questions and to distinguish between overlapping effects.

Pathologic tubulin mutations are further linked to neurodevelopmental and neurodegenerative disorders summarized as tubulinopathies^65^. Among them, eight point-mutations in the *Tuba4a* gene have been associated with ALS in patients^66^, connecting this tubulin isotype to neurodegeneration, however, none of them are located within the C-terminal tail affecting Tuba4a polyglutamylation. The relatively late expression of Tuba4a in neurons (Fig. 2H, I) matches with a potential role in late-onset disease.

Within the family of microtubule-associated proteins, neuronal Tau mainly binds to axonal microtubules. Mechanistically, Tau is thought to regulate the long labile domains of microtubules^24^, stimulating their growth and inhibiting their shrinkage^25^. Under pathological conditions, hyper-phosphorylated Tau detaches from microtubules and forms aggregates, known as neurofibrillary tangles. However, it is controversially discussed, whether Tau accumulation represents a cause or consequence of tauopathies^1–3^. The binding site between Tau and microtubules and the regulation of their interaction has been characterized by liquid-liquid phase separation in solution using NMR and other biochemical methods^67^. Previous biochemical in vitro data suggested C-terminal tubulin polyglutamylation as a potential regulator of Tau-microtubule interactions^32^. Cryo-electron microscopy further characterized different full-length and truncated Tau constructs with respect to their microtubule affinity^68^. Based on these data, computational modeling predicts that Tau interacts with microtubules through four conserved tubulin-binding repeats that span over three tubulin monomers along a protofilament. Notably, the adjacent C-terminal tail of alpha-tubulin is located in a perfect position to regulate this interaction^68^. In combination with the results of the present study, it is plausible to conclude that Tuba4a polyglutamylation might be a critical determinant in this scenario. However, the loss of Tuba4a C-terminal polyglutamylation also reduced the binding of MAP2a/b to microtubules and both MT-associated proteins, MAP2a/b and Tau, have been shown to regulate microtubule dynamics^24,62^. In line with this, we observed a decrease in microtubule growth in Tuba4aΔpolyGlu derived neurons at the onset of Tuba4a expression. On the other hand, microtubule binding of MAP1 remained unaffected, supporting that polyglutamylated tubulins differentially regulate MAP-MT binding^46^.

Motor protein movement along microtubules can be regulated by tubulin polyglutamylation^33,64^. Accordingly, reduced polyglutamylation, following the loss of TTLL1 gene expression, resulted in higher motility of cargo (mitochondria) and consequently to a degeneration of motor axons^36^. However, the specific loss of Tuba4a C-terminal polyglutamylation described in our study, revealed no effect on KIF5 velocities in dendrites or axons and did not induce neurodegeneration, suggesting that the regulation of kinesin-microtubule interactions might be mediated by specific tubulin isotypes other than Tuba4a. Alternatively, the depletion of TTLL1, which modifies substrates other than tubulin^38–42^, might lead to secondary effects that are presently unknown.

Hyper-phosphorylation is thought to induce Tau detachment from microtubules and its subsequent aggregation into cytosolic neurofibrillary tangles^1,2^. In the present study, our Tuba4aΔpolyGlu mouse was characterized by a major loss of Tau-microtubule binding, however, with Tau remaining in an un-phosphorylated state. This hypo-phosphorylation of Tau differs from pathological conditions characterized by hyper-phosphorylated Tau. Notably, in both settings, Tau binding to microtubules is markedly diminished. According to current knowledge, hyper-phosphorylation of Tau reduces its affinity for microtubules. Subsequently, abnormally hyper-phosphorylated Tau misfolds, leading to self-aggregation into NFTs^1,2^. In the present study, analyzing hTau mice at one year of age, we detected oligomerization of hyper-phosphorylated Tau, but not yet aggregation of the protein. Consistent with the late onset of AD-like Tau pathology in this particular hTau mouse model^69^, these observations likely reflect early disease conditions. In addition, reduced hTau expression due to a shortening of the transgene array over nineteen years of breeding might contribute to this effect^70^.

GSK3 is one of the major kinases to phosphorylate Tau and represents a risk factor in AD^71–73^. Under physiological conditions, GSK3 is directly associated with neuronal microtubules in a phosphorylation-complex containing Tau^74,75^. The present study supports and extends this view, suggesting that Tuba4a polyglutamylation is critical to recruit GSK3 and Tau to microtubules, representing a prerequisite for efficient Tau phosphorylation. In turn, under pathological conditions, a lack of Tuba4a polyglutamylation (hTau/Tuba4aΔpolyGlu mouse in Figs. 5–8), might be protective, as the phosphorylation of Tau through GSK3 becomes inefficient, thereby precluding hyper-phosphorylation and consequently Tau oligomerization. This link however needs to be further investigated, since under physiological and pathological conditions, Tau is phosphorylated at different epitopes through a variety of kinases other than GSK3^76^. For instance, the microtubule-affinity regulating kinases (MARK), which control the dynamics and polarity of the microtubule cytoskeleton, are involved in the regulation of Tau/MAP-microtubule binding^77^. Interestingly, MARK2 is directly phosphorylated by GSK3β, thereby inhibiting its activity^78^. Future investigations will also have to clarify the role of isotype-specific tubulin polyglutamylation, with respect to Tau, Tau-specific kinases, and Tau-microtubule interactions in physiological and disease conditions.

To gain a better understanding of the role of Tau in disease, many transgenic or knockout mouse models have been developed and studied over the years^79^. Notably, a classical gene knockout of Tau revealed no severe phenotype^80,81^, indicating that a functional loss of Tau is tolerated and most likely compensated through other MAPs. Our data, showing severely reduced Tau binding to microtubules without major developmental of functional deficits in the Tuba4aΔpolyGlu mouse, support this view. They suggest that the Tau phosphorylation state, which functionally depends on Tuba4a polyglutamylation, represents a critical parameter in disease progression and therefore can be reversed in the hTau/Tuba4aΔpolyGlu mouse model. However, since Tuba4a expression in neurons is relatively late, these effects seem to be most critical at adult physiological and pathophysiological conditions. In contrast, the phosphorylation of Tau, during early development^82^ might be regulated by mechanisms independent of Tuba4a polyglutamylation.

One of the early signs of neurodegeneration is the activation of microglia, which together with other glial cells, leads to a pathological condition, known as gliosis^83^. In the healthy brain, microglia display a basal motility of their processes without moving their cell bodies. Neuronal damage activates microglia, leading to their proliferation with an increased mobility and extension of their processes that rapidly phagocytose harmful material^84^. Synapse loss and microglial activation are accompanied by tangle formation in Tau pathology^85^ and microglia remove synaptic compartments of neurons with Tau pathology^86^. In addition, microglia activation might promote the propagation of Tau aggregates, leading to negative feedback^87^. Other studies that employed the hTau mouse model by Andorfer and colleagues^51^, reported gliosis prior to neurodegeneration^51,88^. In line with the data of the present study, 5XFAD mice, representing an alternative AD mouse model^89,90^, are characterized by a specific loss of layer 5 neurons, thereby connecting Tau hyperphosphorylation/oligomerization and activated microglia within cortical layer 5 (compare with Supplementary Figs. 8 and 11). Furthermore, pyramidal cells in layer 5 are characterized by high levels of amyloid beta^91^, supporting the view that this cortex layer might be highly vulnerable.

Remarkably, the number and activity state of microglia in the present study is normalized in hTau/Tuba4aΔpolyGlu mice, as compared to hTau mice. Although we cannot exclude parallel Tuba4a expression by microglia, this indicates at the in vivo level that the loss of Tuba4a C-terminal polyglutamylation not only protects against Tau hyper-phosphorylation and its oligomerization, but further normalizes microglia activation.

Other studies linked increased polyglutamylation to neurodegenerative disease^42,64,92–94^. In addition, the presented data extend this view, showing that the loss of polyglutamylation at a specific tubulin isotype, protects against Tau pathology and microglia activation. Although subcellular consequences with respect to microtubule dynamics or transport remain to be investigated, our data highlight an important subcellular mechanism that has the potential to develop treatment strategies in fighting tauopathies.

## Methods

### Animals

Animals were maintained in the animal facility of the ZMNH, Hamburg (Germany) under controlled environmental conditions. Mice (both sexes were used for experiments) were group-housed (2–5 mice per cage) under a 12-hour light/dark cycle. Temperature (22 ± 1 °C) and humidity (50 ± 5%) in the animal facility were kept constant, and the animals had ad libitum access to food and water. All animal experiments complied with all ethical regulations for animal testing and research in accordance with the European Communities Council Directive (2010/63/EU) and were approved by the ethics committees of the city-state of Hamburg (Behörde für Justiz und Verbraucherschutz, Fachbereich Lebensmittelsicherheit und Veterinärwesen (reference (ID number) 100/13) and the animal care committee of the University Medical Center Hamburg-Eppendorf.

### Generation of Tuba4aΔpolyGlu mice

To generate the Tuba4a knock-in mouse, a Cre-activated allele approach was used. Based on Tuba4a cDNA sequence NM_009447 the exon/intron organization of the gene was established. The following mutations were introduced: E433D, E434D, E441D, E443D, E445D, E447D, and E448D. A targeting vector was generated and used for homologous recombination in embryonic stem (ES) cells to modify the endogenous *tuba4a* gene (constructed by GenOway, Lyon, France) (Supplementary Fig. 1C). ES cell clones were screened by Southern blot analysis. Chimeric males were obtained by injection of positive clones into C57BL6/J 8-cell stage embryos and were mated with C57BL6/J wild-type female mice to establish germline transmitted founders. Heterozygous offspring with a minimum of three directed backcrosses against the C57BL6/J background to eliminate mutations in Crb1 (Rd8-mutation), Cyfip2, and Nnt, were intercrossed to obtain wild-type and homozygous knock-in animals. To remove the FRT-flanked neomycin selection cassette, these mice were mated with FLP-Deleter (ACTB-FLPe) mice (Jackson Laboratory, Bar Harbor, ME, USA, #005703)^95^ to obtain the floxed allele used in this study. Subsequent mating with CMV-Cre mice (Jackson Laboratory, #006054)^96^ employed Cre/loxP recombination for genome-wide induction of the mutated *tuba4a* gene. To investigate the effect of Tuba4a-hypo-glutamylation on human Tau phosphorylation and localization, heterozygous mutated *tuba4a* knock-in mice were crossed with hTau mice (Jackson Laboratory, #005491)^51^, an established mouse model for tauopathies, expressing all six isoforms of human Tau instead of mouse Tau.

### Protein modeling

The protein structure of mouse Tuba4a (P68368) and mouse Tubb2a (Q7TMM9) (Fig. 1B–C) was modeled using the protein structure homology-modeling server SWISS-MODEL (swissmodel.expasy.org).

### Long range PCR

To validate the correct targeting of the mutated *tuba4a* knock-in, long-range PCR (Supplementary Fig. 1F) was performed by using 2 oligonucleotides (0066-Neo ATGCTCCAGACTGCCTTGGGAAAAG (I), 70246sa: CAGAACCACAGGATGTCTCCACAACC (II)). The resulting 3582 base pair product was sub-cloned into a pGEM-T Easy-Vector (Promega, Madison, WI, USA, #A1360) and was validated by Sanger sequencing analysis.

### Genotyping

Genomic DNA was isolated from tail biopsies using the Quick Extract Buffer (Biozym Scientific GmbH, Hessisch Oldendorf, Germany, #101098). For genotyping of mutated *tuba4a* knock-in mice, 2 oligonucleotides were used (70252hom: GAGGAGGGAGCTTTGGACTCTGTGC (III); 70253hom: AGCAGAAAGGGGGACAAGCAGAGG (IV)). Expected PCR product sizes: 1302 base pairs for the wild-type allele and 1440 base pairs for the knock-in allele. For genotyping of hTau mice 3 oligonucleotides (dRTau_for: CAGGCTTTGAACCAGTATGG; dRTau_rev: TGAACTTGTGGCCGTTTACG; mTau_2rev: CTAAGGACTGCTGTAGAACTG) were used to analyze the mouse *Tau* knockout-locus. Expected PCR product sizes: 383 base pairs for the wild-type allele and 170 base pairs for the knockout allele. The human *Tau* knock-in locus was analyzed using the following 4 oligonucleotides: hTau_3s: GATGTAAATAACGCTTGGGCAGGAATA; hTau_rev: CTGTGCATGGCTGTCCCTACCTT; non_hTau_as: GTACATCGGATTAGCAAAAGGAAGAC and hTau_2s: CATTGTACTTTTGTGCCCAGACTCAG. Expected PCR product sizes: 273 base pairs for the wild-type allele and 353 base pairs for the knock-in allele.

### Southern blotting

Southern blot analysis was used to confirm correct 5′ and 3′ homolgous recombination events. For the 5′-end, genomic DNA was digested using NsiI and an internal probe (346 bp) located within the 5′-homology arm of the targeting vector was expected to detect the following DNA fragments: 12128 bp (wild type) and 8548 bp (recombined). The following oligonucleotides were used to generate the 5′probe: 70244PRO (GGGGAAGGGAGAGAGGTGTATGAAGG) and 70145PRO (CCTGGCAACTTAAGGCTACATCTAGGTGG). For the 3′-end, genomic DNA was digested using AflII and an internal probe (498 bp) located within the 3′-homology arm of the targeting vector was expected to detect the following DNA fragments: 5789 bp (wild type) and 6766 bp (recombined). The following oligonucleotides were used to generate the 3′probe: 70242PRO (GGCTGCCCCTCATCACCAAGC) and 70243PRO (TGGGGCTCAGAGGACTATAGGGTCC).

### Antibodies

The used primary antibodies are summarized in Supplementary Table 1, the secondary antibodies in Supplementary Table 2.

### Histology and immunohistochemistry

For Nissl staining adult mice were euthanized with CO_2_ and perfused using 4% PFA/PBS (w/v). Brains were harvested and post-fixed for 6 h in 4% PFA/PBS. A 30% Sucrose/PBS (w/v) solution was used for dehydration. Brains were then embedded in Tissue Tek (SakuraTek, Ca, USA, #4583) and frozen. 30 μm coronal sections were cut using a cryostat (CryoStar, Thermo Fisher Scientific, Waltham, MA, USA) and conserved at −80 °C.

For Cresyl violet staining, 30 μm sections were sequentially rinsed in 70% (v/v), 95% (v/v), 100% ethanol and xylene. Slices were rehydrated by performing the washing row backwards with a final washing step in water and were subsequently incubated for 3 min in Cresyl violet. Following a 1 min wash in water, sequential incubations in 70% ethanol (v/v), 95% acid ethanol (acetic acid), 95% ethanol (v/v), 100% ethanol and Xylene were performed. Sections were then mounted with Entellan (Sigma-Aldrich, St. Louis, MO, USA, #107906).

For immunohistochemistry, brains were harvested and post-fixed in 4% PFA/PBS (w/v) for 48 h followed by a wash in PBS for 24 h. Brains were subsequently dehydrated by incubation in the following solutions for 1 h each at 30 °C: 50% (v/v) ethanol, 70% (v/v) ethanol, 95% ethanol (v/v), 2 × 100% ethanol. After dehydration brains were immersed in three changes of Roti Histol (Carl Roth, Karlsruhe, Germany, #6640.1) for 30 min each at 30 °C, followed by three changes of Paraplast X-TRA (Leica, Nußloch, Germany, #39603002) for 2 h each at 60 °C, before being embedded in Paraplast X-TRA. Brains were cut to 5 µm sections using a rotary microtome (Leica RM 2165) and were stored at room temperature (RT) until staining was performed. Before staining, sections were sequentially rehydrated in Roti Histol, 50% Roti Histol (in ethanol, v/v), 100%, 95% (v/v), 70% (v/v) and 50% (v/v) ethanol, followed by a brief wash in tap water. Permeabilization was performed by incubation for 10 min each in TBS containing 0.1% Triton X- 100 (v/v). Unspecific epitopes were blocked for 1 hour in TBS containing 10% goat serum (v/v), and 0.1% Triton X-100 (v/v). Primary antibodies were incubated over night at 4 °C diluted in antibody incubation buffer (PBS, 3% goat serum (v/v), 0.1% Triton X-100 (v/v)). The following day, sections were washed three times and incubated for one hour with fluorescently tagged secondary antibodies in antibody incubation buffer. Optionally antigen retrieval was performed for 15 min at 95 °C in Na-citrate Buffer (10 mM, pH 6.0). Nuclei were visualized with DAPI (Merck, Darmstadt, Germany; #124653, ICC/IHC 1:1000). Sections were then mounted in Aqua Poly/Mount (Polysciences, Warrington, PA, USA, #18606) and dried overnight in the dark.

### Behavioral experiments

Mice (male and female; both sexes used for all experiments) were group-housed (2–5 mice per cage) under a reversed 12-h light/dark cycle. Temperature (22 ± 1 °C) and humidity (50 ± 5%) in the animal facility were kept constant, and the animals had ad libitum access to food and water. The behavioral experiments were conducted during the dark phase of the light cycle. The composition of the cohort was the following: 28+/+ mice; 13+/− mice and 21−/− mice. The behavioral testing sequence was the following: open field test (1 day), accelerating rotarod (3 days).

#### Open field test

The apparatus consists of four identical arenas (50 × 50 × 40 cm) made of PVC foam material with a white floor and individually illuminated by a lightning bulb (70 lux). The locomotor activity was monitored online by a video camera coupled to a computer using the Ethovision tracking system (version XT 8.5, Noldus Technology, Wageningen, The Netherlands). Animals were placed in the center of the open field and were allowed to freely explore the arena for 60 min. At the end of each trial, mice were returned to the home cage and the arena was cleaned. Total distance traveled in 60 min and total distances traveled within 10-min bins were used as measures for locomotor activity.

#### Accelerating rotarod test

Mice walked on a rotating rod (Acceler, Rotarod for mice, Jones & Roberts, TSE Systems, Bad Homburg, Germany) at constant speed (5 rpm) for two minutes. After two acclimatization trials on the first day of testing, each mouse was placed on the rotating rod for two test trials on the second day, during which the rotation speed gradually increased from 4 to 40 rpm within four minutes, followed by an additional minute at constant rotation speed of 40 rpm. On the third day, each mouse was placed on the rotating rod for three test trials, during which the rotation speed gradually increased, according to the second day. The inter trial interval (ITI) was always 1 h. Performance was evaluated by measuring the latency to fall for the individual trails by applying accelerating rotation speed on the second and third day of testing.

### Ex vivo protein extracts

To quantify the individual protein expression levels, the brains of adult mice were dissected in PBS on ice as previously described^19^. The tissue was immediately suspended in lysis buffer (PBS, containing 1% (v/v) Triton X‐100 (Sigma-Aldrich, #T8787), PhosSTOP phosphatase inhibitor (Roche, Basel Switzerland, #4906845001), Complete protease inhibitor (Roche, #4693116001), and 1 mM PMSF and homogenized by vigorous shearing of the tissue with five strokes of a 1 ml syringe equipped with a 21 (first stroke) and 26 (remaining strokes) gauche canula. The homogenate was incubated for 30 min on ice and subsequently centrifuged at 1000 × *g* for 10 min at 4 °C (S1 extracts). Alternatively, to extract heat-stable proteins, homogenates were boiled for 10 min prior to centrifugation for 10 min at 20,000 × *g*. The resulting supernatants were boiled in SDS sample buffer (0.0625 mM Tris pH 6.8, 2% SDS (v/v), 0.1 M DTT, 10% glycerol (v/v), 0.01% (v/v) bromophenol blue). Alternatively, to keep proteins in a semi-native state, boiling in SDS sample buffer was omitted. Ten micrograms of total protein per sample were subjected to SDS–PAGE and subsequently analyzed by western blotting.

### Western blotting

For detection of relative immunoblot signal intensities, images were acquired using a Chemo‐Cam Imager ECL HR 16‐3200 (Intas). Alternatively, images were acquired using an Odyssey CLx (LICOR) imaging system. Signal intensities were analyzed using Fiji (ImageJ, version 2.0, NIH, USA) or Image Studio Lite (version 5.2, LICOR). Total protein staining was performed using Revert 700 Total Protein Stain (LICOR, NE, USA, #926-11021), according to the manufacturer’s instructions.

### Immunoprecipitation

For co-immunoprecipitation experiments, hippocampi of adult Tuba4a (+/+) wild-type and (−/−) knock-in mice were dissected in ice cold PBS, homogenized in IM-Ac buffer (20 mM HEPES, 100 mM KAc, 40 mM KCl, 5 mM EGTA, 5 mM MgCl_2_, pH 6.75) with freshly added Complete protease inhibitor, 1% (v/v) Triton X‐100, 1 mM PMSF, 5 mM DTT and 2 mM Mg-ATP and incubated for 30 min on ice. All following steps were performed at 4 °C. The homogenate was clarified by centrifugation at 1000 × *g* for 10 min and the resulting supernatant (S1) was precleared by incubation with magnetic Protein G Dynabeads (Invitrogen, Carlsbad, CA, #1004D) for 20 min. After coupling of 2 µg rabbit anti-alpha4a-tubulin antibodies to magnetic Protein G Dynabeads, precleared extracts from S1 were incubated with beads for 2 h, followed by extensive washing steps with IP-buffer (150 mM NaCl, 50 mM Tris, pH 6.75, 5 mM MgCl_2_). Bound proteins were eluted in SDS sample buffer, subjected to SDS-PAGE and subsequently analyzed by western blotting.

### Cell culture, transfection, tissue extraction, and immunocytochemistry

Primary hippocampal neurons were prepared as previously described^19^. Briefly, one day prior to the preparation, 12 mm sterile glass coverslips were placed into 24‐well plates and coated with poly‐l‐lysine (50 μg/ml) over night at 37 °C. Coverslips were rinsed twice with 500 μl sterile H_2_O. About 1 ml of preheated complete neurobasal medium (Thermo Fisher Scientific, #A3582901) supplemented with l‐glutamine (Thermo Fisher Scientific, #25030024) and B‐27 (Thermo Fisher Scientific, #A3582801) was added to each well. Embryos at stage E 15 were dissected in PBS containing 2 mM glucose at 4 °C. Hippocampi of C57BL/6 mice were dissected, transferred to 1 ml 0.05% trypsin/EDTA (v/v) solution and incubated for 5 min at 37 °C. After removal of trypsin, the hippocampi were washed with DMEM‐F12‐containing serum (Thermo Fischer Scientific, #11320074) and kept in HBSS medium (Thermo Fisher Scientific, #14065056). Hippocampi from both sexes were gently dissociated into cells, using fire polished Pasteur pipettes. Cell density was calculated using a Neubauer Counting Chamber (Marienfeld, Lauda‐Königshofen). 60,000 cells were plated per well in 24‐well plates and kept at 37 °C in a humidified atmosphere of 95% air and 5% CO_2_ in a cell incubator. Cells cultured for 10–11 days in vitro (DIV) were used for transfection by a calcium phosphate co-precipitation protocol^97^. About 16 h after transfection cells were fixed. To quantify the protein expression levels of tubulin in primary hippocampal neurons, cultures were harvested after 1, 3, 7, 14, or 20 days in vitro (DIV), respectively as previously described^19^. The adherent cells were washed once in ice‐cold PBS and harvested in PBS lysis buffer, containing 1% (v/v) Triton X‐100, PhosSTOP phosphatase inhibitor, Complete protease inhibitor, and 1 mM PMSF. After incubation for 30 min on ice, lysates were centrifuged at 1000 × *g* for 10 min at 4 °C (S1 extracts). The resulting supernatants were boiled in SDS sample buffer after adjustment of protein concentrations using a BCA assay (Pierce Biotechnology, Rockford, IL, USA, #23225). About 3 µg of total protein per sample per time point (days in vitro) were subjected to SDS–PAGE. Subsequently, samples were analyzed by western blotting.

For confocal laser-scanning microscopy analysis, cultured hippocampal neurons were fixed in 4% paraformaldehyde (w/v)), 4% sucrose (w/v) diluted in PBS for 12 min. Cells were permeabilized for 4 min in PBS containing 0.25% Triton-X-100 (v/v), and blocked in PBS containing 1% (w/v) BSA. Neurons were incubated for one hour in primary antibodies diluted in blocking buffer at RT or overnight at 4 °C. After a wash step, secondary antibodies and optionally DAPI were incubated for one hour at RT. Coverslips were mounted in Aqua Poly/Mount.

For the analysis of microtubules and STED imaging, an optimized protocol was used as previously described^98^. Briefly, cells were rinsed in pre-warmed HEPES buffer (10 mM HEPES (pH 7.4), 135 mM NaCl, 5 mM KCl, 2 mM CaCl_2_, 2 mM MgCl_2_, 5 mM glucose) pre-extracted and fixed in extraction buffer containing 80 mM PIPES (pH 6.9; KOH), 7 mM MgCl_2_, 1 mM EGTA, 0.3% (v/v) Triton-X100, 150 mM NaCl, 5 mM glucose, 0.25% glutaraldehyde (v/v) for 90 s at 37 °C and then in PBS with 4% PFA (w/v) and 4% (w/v) sucrose for 10 min at 37 °C. After fixation, cells were washed three times in PBS and cells were further permeabilized for 10 min in PBS with 0.25% (v/v) Triton-X100. Cells were then washed three times in PBS, quenched for 1 h with blocking buffer (50 mM NH_4_Cl in PBS, 2% (w/v) BSA, 0.2% (w/v) gelatin, 10 mM glycine), washed again, and incubated with the primary antibody diluted in blocking buffer over night at 4 °C. For the secondary antibody labeling, coverslips were washed from the primary antibody and anti-mouse or ant-rabbit antibodies conjugated to AF647 or AF594 were diluted in a same blocking buffer and added for 1 h at RT. Subsequently, coverslips were washed with PBS, post-fixed in PBS with 2% (w/v) PFA and 2% (w/v) sucrose for 10 min at room temperature, again extensively washed with PBS and mounted for imaging.

### Confocal laser-scanning and STED imaging

For confocal microscopy, imaging was carried out as described before^99^. Briefly, for imaging of fluorescent signals, a Laser-Scanning Confocal Microscope Fluoview FV1000 (Olympus Hamburg, Germany) equipped with a 60× objective and Fluoview software version 2.1b was used. For multichannel fluorescent imaging, images were sequentially recorded using identical photomultiplier values throughout all individual experiments. Experiments were replicated at least three times from individual preparations of tissue. Images were analyzed using the MetaMorph software version 6.3 r7 (Universal Imaging, Downingtown, PA, USA). ROIs were defined by the “ROI tool function” throughout multiple frames. Overlay files were separated using the “color separate” function within the software package. Identical ROIs were selected using the “transfer region” function. Brightness was adjusted with the “inclusive thresholding” function to define image thresholds. For measurements of fluorescence intensity, the integrated “morphometry analysis” function was used to assess total and average signal intensities of identical ROIs for each individual channel. For stimulated emission depletion (STED) super-resolution microscopy, imaging was carried out as previously described^100^. Briefly, STED images were acquired in gating mode by mean of an Abberior expert line laser scanning STED microscope. As excitation, two pulsed laser sources operating at 561 and 640 nm were used while as depletion beam a short-pulsed 775 laser was employed. For excitation and detection, a 60X NA = 1.4 P-Apo Oil objective from Nikon was used. Images were taken at different zoom factors setting to pixel size to be 20 nm in *x* and *y* (the number of total pixels varying depending on the scanned image area). Images were acquired at constant scanning speed (pixel dwell 3 s/µm) and accumulating multiple lines.

### Line scan analysis

Line scans were applied using the “Plot profile” tool of Fiji (ImageJ, version 2.0, NIH, USA), to measure signal intensities and identify signal peaks of individual STED channels along a defined region of interest. To assess co-localization, the distance between signal peaks was analyzed.

### Immunogold labeling and transmission electron microscopy analysis

Adult male mice were terminally anesthetized with sodium pentobarbital (1.6 mg/g body weight, Narcoren, Boehringer Ingelheim, Germany). Perfused brains were isolated and stored in a buffer containing 3% PFA (w/v), 220 mM saccharose, 16 mM KH_2_PO_4_, and 84 mM Na_2_HPO_4_ × 2H_2_O at 4 °C for 24 h. Sagittal vibratome sections of 70 μm thickness of the medulla oblongata (rich in parallel axons) were used for pre-embedding immunoelectron microscopy. In brief, the vibratome sections were blocked in 2% normal goat serum (v/v) at RT for 30 min and then incubated with a rabbit monoclonal Tubulin-alpha4a-antibody diluted in PBS at 4 °C for 24 h. Following several rounds of washing with PBS, incubation with a secondary goat anti-rabbit antibody conjugated with nanogold particles of a diameter of 10 nm was carried out at RT for 2 h. Sections were washed in PBS several times and fixed with 1% aqueous glutaraldehyde (v/v) at room temperature for 20 min. Sections were then rinsed in distilled water, washed several times in an aqueous solution of 0.8% NaCl (w/v) and 20% sucrose (w/v) at RT for 30 min, and osmicated in aqueous 2% OSO_4_ (w/v) at RT for 2 h. The osmicated sections were dehydrated in ascending concentrations of ethanol and then incubated in propylene oxide for 1 h prior to embedding in propylene oxide: Araldite (1:1, 2 h at room temperature) and pure Araldite resin containing 2% accelerator. Ultrathin sections of 70 nm thickness of the fiber tracts traversing the medulla oblongata were cut and subjected to low-voltage electron microscopy at 25 kV (LVEM25, Delong Instruments, Brno, Czech Republic).

### Live cell imaging

For time-lapse imaging, coverslips with the transfected neurons were mounted into a live-cell imaging chamber containing a conditioned medium and kept at 37 °C and 5% CO_2_ levels in an incubator coupled to the spinning disk microscope. To observe the transport of the motor protein KIF5c, neurons at DIV12 were transfected with KIF5c-tdTomato-pex26 and analyzed 24 h later (frame rate: 1 frame/second; duration: 200 s). In order to examine microtubule dynamics, neurons at DIV3, 4, and 11 were transfected with the microtubule plus-end marker EB3-GFP and imaged 24 h later. Only EB3 comets that appeared and disappeared during a time period of 300 seconds (frame rate: 0.5 frames/second) were included in the analysis. Time-lapse images of transfected neurons were acquired using a Nikon spinning disc microscope (Visitron, Puchheim, Germany) equipped with a 60× (for KIF5c imaging) and 100× (for EB3 imaging) objective and 488-nm and 561-nm argon lasers. For the final analysis, captured LSM images were exported as TIF images using VisiView (VisiView 4.0, Visitron, Puchheim, Germany). Anterograde and retrograde KIF5c single-particle mobility, as well as EB3-tdTomato comet growth of sequential images within dendrites were identified based on their morphology (presence of mushroom-type protrusions) and axons were quantified manually using Fiji (ImageJ, version 2.0, NIH, USA). Background correction was achieved through subtraction of average intensity. Kymographs of dendritic segments were generated using the Multiple Kymograph plug-in for Fiji. All immunofluorescence quantifications were performed on the MetaMorph 6.3 r7 software or using Fiji.

### Soluble-tubulin extraction assay (TX-100 extraction)

To separate soluble tubulin, dissociated microtubule (MT), and dissociated proteins (e.g., MAPs) from the insoluble cytoskeleton with bound/associated MAPs, primary hippocampal neurons at DIV 15 were extracted using a detergent-containing buffer^98,101^. Therefore, 80,000 cells were seeded in a 24-well size vessel and cultured for the indicated time. All following steps were performed at 37 °C. Cells were briefly washed with HEPES buffer (10 mM HEPES (pH 7.4), 135 mM NaCl, 5 mM KCl, 2 mM CaCl_2_, 2 mM MgCl_2_, 5 mM glucose) and subsequently incubated for 90 s in 300 µl microtubule-extraction buffer (MT-extraction buffer; 80 mM PIPES, 7 mM MgCl_2_, 1 mM EGTA, 0.3% (v/v) Triton X-100, 150 mM NaCl, 5 mM glucose, pH 6.9 (KOH)). The extract was entirely removed and supplemented with a final concentration of 1% (v/v) Triton X-100 (soluble cytosolic fraction). The remaining tissue in the culture plate was harvested in 300 µl MT-extraction buffer supplemented with a final concentration of 1% (v/v) Triton X-100 using a cell-scraper and homogenized using a 200 µl-pipette (in-soluble cytoskeletal fraction). After incubation for 30 min on ice, both extracts were centrifuged at 1000 × *g* for 10 min at 4 °C. Resulting supernatants were boiled in SDS sample buffer after adjustment of protein concentrations using a BCA assay. Fifteen micrograms of total protein per sample were subjected to SDS–PAGE and subsequently analyzed by western blotting.

### Endogenous microtubule preparation from adult mouse brain

Protocol according to the Mitchison lab, Harvard University (http://mitchison.med.harvard.edu/Protocols.htm). To polymerize microtubules from mouse brain and to analyze the fraction of attached MAPs, adult mice were scarified and the hippocampus was dissected in ice-cold PBS. The tissue was homogenized in 1.3 ml BRB80 buffer (80 mM PIPES, pH 6.8 (KOH), 1 mM MgCl_2_, 1 mM EGTA, 1 mM DTT) by vigorous shearing of the tissue with five strokes using a 1 ml syringe equipped with 26 gauche canula followed by 8 strokes at 900 rpm using a Teflon-plunger (Sartorius AG, Göttingen, Germany). The homogenate was centrifuged at 70,000 × *g* for 20 min at 4° (rotor: TLA100.3, Beckman Coulter GmbH, Krefeld, Germany). Afterwards, the supernatant was removed, supplemented with 1 mM GTP and 2 mM ATP and incubated for 10 min at RT. Subsequently, the homogenate was divided into three parts supplemented with: (i) 10 % (v/v) DMSO kept at 37 °C for 30 min, (ii) 2 mM Taxol (Sigma-Aldrich) 5 mM Taxol kept at RT for 3 min, followed by 30 min at 37 °C and (iii) 5 mM CaCl_2_ stored on ice for 30 min. In addition, all parts were supplemented with a complete protease inhibitor. Afterwards, the samples were transferred on top of a pre-warmed BRB80 cushion containing 40% (v/v) glycerol and centrifuged at 200,000 × *g* at 37 °C for 30 min. The resulting supernatant (containing soluble tubulin dimers and detached MAPs) was removed and kept on ice. The resulting pellet (containing polymerized microtubules and attached and co-pelleted MAPs) was resuspended in 8 M urea over 30 min at RT. Finally, fractions were boiled in SDS sample buffer and a fixed volume of total protein per sample were subjected to SDS–PAGE and subsequently analyzed by western blotting.

### Analysis of mRNA expression levels

RNA extraction and quality control: RNA extraction was performed using Trizol (Invitrogen, #15596026), with one hippocampus from wild-type or transgenic animals aged 12 or 18 weeks, respectively, as starting material. The samples were homogenized by vigorous shearing of the tissue in 1000 µl Trizol with 10 strokes of a 1 ml syringe equipped with a 20 and 23 gauche cannula, succeedingly. Total RNA was photometrically quantified by NanoDrop 2000 (Thermo Fisher Scientific) measurement. Moreover, ~500–1000 ng RNA were analyzed on a 1.2% agarose gel (w/v) with a 1-Kb marker for overall RNA quality control. Reverse transcription was performed using 5 µg total RNA, oligo dT primers, and hexamer primers of the Superscript Reverse Transcription Kit (Thermo Fisher Scientific, #18064071).

Quantitative PCR (qPCR) analysis: qPCR reactions were performed as duplex assays with the Taqman gene expression master mix (Thermo Fisher Scientific, #4369542). Primers were used with purification grade desalted. qPCR reactions were performed on Applied Biosystems 7900 HT Fast Real-Time PCR System (Applied Biosystems, Foster City, CA, USA) in a 96-well format. qPCR reaction volumes were 20 µl, number of replicates per sample was four. Thermal cycling conditions: activation of HotStarTaq DNA Polymerase at 95 °C for 15 min and two-step cycling with denaturation at 94 °C for 15 s, annealing and extension at 60 °C for 30 s, and fluorescence data collection run in 40 cycles. For the initial characterization of the *Tuba4a* forward and reverse primers a melting curve was performed. After incubation at 95 °C for 15 s, the melting analysis was performed from 60 to 95 °C with a 2% ramp rate. Normalization of qPCR-based mRNA expression analysis was performed with the Mm99999915_g1 taqman gene expression assay for GAPDH, primer limited, Vic labeled (Thermo Fisher Scientific) as reference. The primers for *Tuba4a* were: base 217 to base 306, Accession: NM_009447, 85-for-a4a: 5′- CTG TGA AAC TGG AGC TGG AA, 85-rev-a4a: 5′- TAT GGG CCA TTT CGG ATC T; *Tuba4a* probe was mouse Universal ProbeLibrary probe: #85, cat.no. 04689097001, 5′-FAM-labeled (UPL, ROCHE). Differential gene expression was calculated according to the Pfaffl delta-delta Ct-Method, the REST software (REST-2009, gene-quantification, TU-Munich, Germany) was used for the evaluation of the significance of the relative differences^102^.

### De-phosphorylation of protein extracts

To analyze proteins independent of posttranslational phosphorylation, the tissue was immediately suspended in lysis buffer (PBS, containing 1% (v/v) Triton X‐100, Complete protease inhibitor) and homogenized by vigorous shearing of the tissue with five strokes of a 1 ml syringe equipped with a 21 (first stroke) and 26 (remaining strokes) gauche canula. The homogenate was incubated for 30 min on ice and subsequently centrifuged at 1000 × *g* for 10 min at 4°. After adjustment of protein concentrations using a BCA assay, the homogenate was treated with Lambda Protein Phosphatase (Lambda PP; New England Biolabs, Ipswich, MA, #P0753) according to the manufacture's protocol. Briefly, 50 µg protein for each sample was incubated for 45 min on 30 °C in the provided buffer supplemented with 1 M MnCl_2_ and 400U Lambda PP. Samples were boiled in SDS sample buffer and 10 µg of total protein per sample were subjected to SDS–PAGE and analyzed by western blotting.

### Virus production and transduction

The adenovirus-vector encoding the CFP-hTau40 fusion protein was provided by E. Mandelkow^103^. *hTau40* codes for the longest human Tau isoform. For stepwise amplification and purification of high-titer recombinant adenoviruses, the protocol by Luo et al. was used^104^. Production and titration of virus particles were performed by the Hamburg Center for Experimental Therapy Research (HEXT) Vector Core Unit, University Medical Center Hamburg-Eppendorf, Hamburg, Germany. For transduction of primary hippocampal neurons at DIV19, the adeno-vector encoding CFP-hTau40 fusion protein and an empty control-vector, respectively, were added to the conditioned growth media. 20 µl virus suspension with a titer of 2.02 × 10^10^ TU/ml were used per 24-well. Forty-eight hours later, the cells were harvested.

### Dot blot analysis

Forty micrograms of ex vivo protein extracts were applied onto nitrocellulose strips (Bio-Rad, Feldkirchen, Germany, 1620115) and air-dried for 15 min. Strips were blocked in 5% nonfat dried milk in TBST for 1 h at RT and incubated with 1° antibody overnight with rotation. Subsequently strips were washed for 3 × 5 min with TBST at RT and then incubated with fluorescent 2° antibodies for 1 h at RT with rotation. After washing for another 3 × 5 min in TBST at RT, signal intensities were detected using the Odyssey CLx (LICOR) imaging system.

### Sholl analysis

For a manual Sholl analysis on each individual cell, existing images were converted to grayscale using Adobe Photoshop. An initial circle of 12 µm diameter was drawn to exclude the soma, followed by concentric circles with increasing radii of 6 µm per circle. Intersections between Iba1-positive microglial branches and each increasing circle were counted.

### Statistics and reproducibility

At least three biologically independent repeats were conducted for each experiment. Statistical analyses were performed with either SPSS (Chicago, IL, USA) or Prism (GraphPad Software Inc., CA, USA). Briefly, after an exploratory data analysis to identify outliers, data were checked for normality using Kolmogorov-Smirnov or Shapiro-Wilk tests. To separate the means of normally distributed data, either a two-tailed unpaired Student *t*-test or one-way or two-way ANOVA was used, while Mann-Whitney or Kruskal-Wallis tests were used to analyze nonparametrically distributed data. Graphs were constructed using Excel (Microsoft, Redmond, WA, USA) or Prism. Nonparametric data are shown as box plots. Normally distributed data are shown as bar diagrams and individual data points are shown as dots, if *n* < 10. Significance was defined as follows: *p ≤ 0.05, **p ≤ 0.01, ***p ≤ 0.001. Further statistical details for individual experiments are outlined in the respective figure legends.

### Reporting summary

Further information on research design is available in the Nature Research Reporting Summary linked to this article.

## Supplementary information

Supplementary information

Reporting Summary

## Data Availability

All data supporting the findings of this study are provided within the paper and its Supplementary Information. Any data are available from the authors upon request. Source data are provided with this paper.

## Source data

Source Data
