# Supplementary material for: Disruption of tubulin-alpha4a polyglutamylation prevents aggregation of hyper-phosphorylated tau and microglia activation in mice
Source: Nat Commun. 2022 Jul 20;13:4192. doi: 10.1038/s41467-022-31776-5 (PMC9300677; doi:10.1038/s41467-022-31776-5)
Supplement: Supplementary file 1 — Supplementary information [file 41467_2022_31776_MOESM1_ESM.pdf]

## Supplementary Information

# Disruption of Tubulin-alpha4a polyglutamylation prevents aggregation of hyper-phosphorylated tau and microglia activation in mice

**Torben J. Hausrat, Philipp C. Janiesch, Petra Breiden, David Lutz, Sabine Hoffmeister-Ullerich, Irm Hermans-Borgmeyer, Antonia V. Failla, Matthias Kneussel**

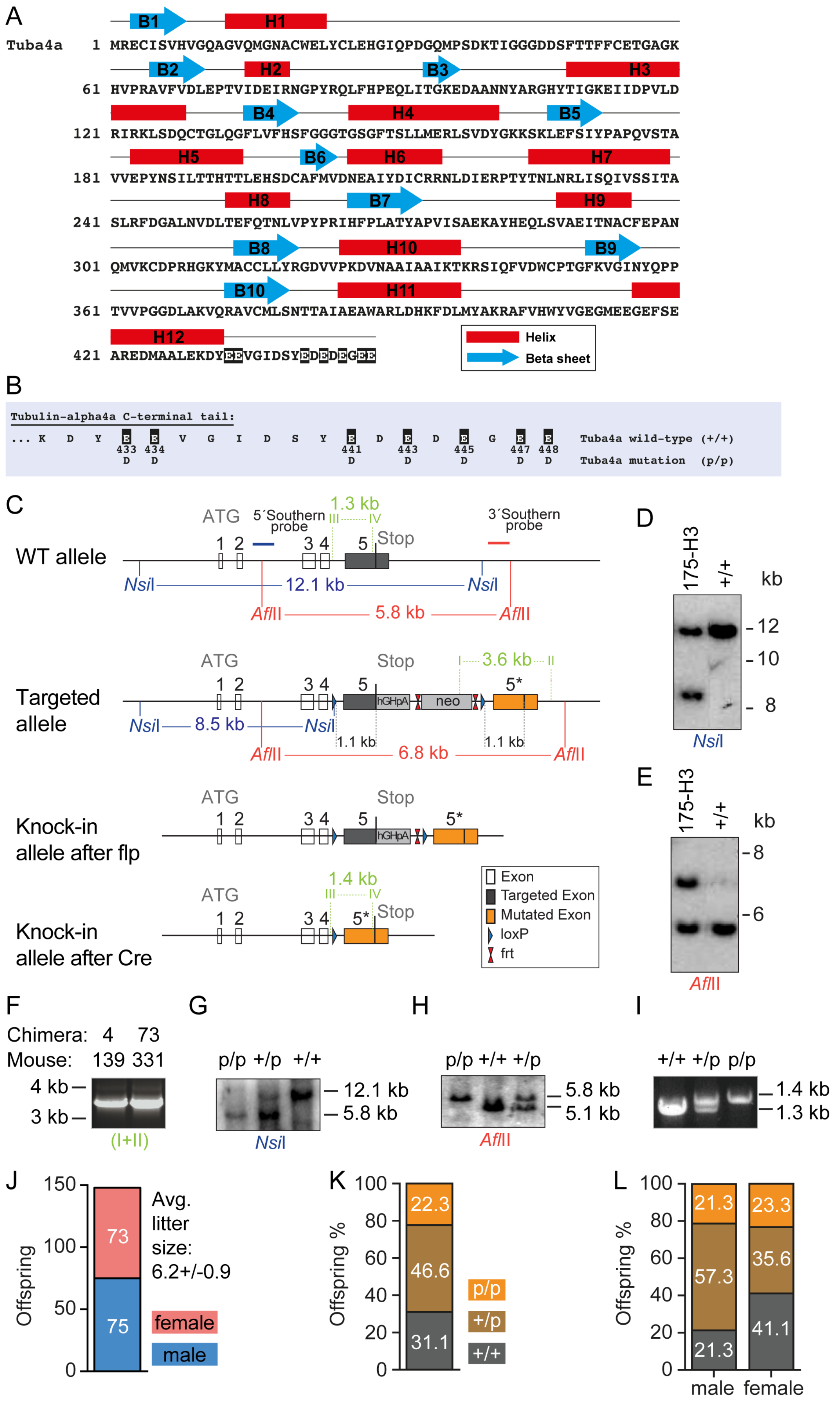

A
B1 H1 B2 H2 B3 H3 B4 H4 B5 H5 B6 H6 H7 H8 B7 H9 B8 H10 B9 B10 H11 H12
Tuba4a 1 MRECISVHVGQAGVQMGNACWELYCLEHGIQPDGQMPSDKTIGGGDDSFTTFFCETGAGK
61 HVPRAVFVDLEPTVIDEIRNGPYRQLFHPEQLITGKEDAANNYARGHYTIGKEIIDPVLD
121 RIRKLSDQCTGLQGFLVFHSFGGGTGSGFTSLLMERLSVDYGKKSKLEFSIYPAPQVSTA
181 VVEPYNSILTTHTTLEHSDCAFMVDNEAIYDICRRNLDIERPTYTNLNRLISQIVSSITA
241 SLRFDGALNVDLTEFQTNLVPYPRIHFPLATYAPVISAEKAYHEQLSVAEITNACFEPAN
301 QMVKCDPRHGKYMACCLLYRGDVVPKDVNAAIAAIKTKRSIQFVDWCPTGFKVGINYQPP
361 TVVPGGDLAKVQRAVCMLSNTTAIAEAWARLDHKFDLMYAKRAFVHWYVGEGMEEGEFSE
421 AREDMAALEKDYEEVGIDSYEDEDEGEE
Helix
Beta sheet
B
Tubulin-alpha4a C-terminal tail:
... K D Y E E V G I D S Y E D E D E G E E Tuba4a wild-type (+/+)
433 434 441 443 445 447 448
D D D D D D D Tuba4a mutation (p/p)
C
WT allele
ATG 5´Southern probe 1.3 kb III IV 3´Southern probe
1 2 3 4 5 Stop
NsiI 12.1 kb NsiI
AflII 5.8 kb AflII
Targeted allele
ATG Stop 3.6 kb I II
1 2 3 4 5 hGHpA neo 5*
NsiI 8.5 kb NsiI 1.1 kb 1.1 kb
AflII 6.8 kb AflII
Knock-in allele after flp
ATG Stop 1 2 3 4 5 hGHpA 5*
Knock-in allele after Cre
ATG 1.4 kb III IV 1 2 3 4 5* Stop
Exon
Targeted Exon
Mutated Exon
loxP
frt
D
175-H3 +/+ kb
12 10 8
NsiI
E
175-H3 +/+ kb
8 6
AflII
F
Chimera: 4 73
Mouse: 139 331
4 kb 3 kb
(I+II)
G
p/p +/p +/+
12.1 kb 5.8 kb
NsiI
H
p/p +/+ +/p
5.8 kb 5.1 kb
AflII
I
+/+ +/p p/p
1.4 kb 1.3 kb
J
Offspring
150 100 50 0
73 75
Avg. litter size: 6.2+/-0.9
female
male
K
Offspring %
100 80 60 40 20 0
22.3 46.6 31.1
p/p +/p +/+
L
Offspring %
100 80 60 40 20 0
21.3 57.3 21.3
23.3 35.6 41.1
male female

**Supplementary Figure 1, related to Figure 1.** (**A**) Primary and secondary structural elements of Tuba4a. Helices (H1-H12) are presented as red rectangles, beta sheets (B1-B10) are presented as blue arrows. Potential glutamylated amino acids within the unfolded C-terminal tail are highlighted in black. Sequence accession number of Tuba4a: NP_033473.1 (**B**) Positions of the point-mutations introduced in Tuba4a. Note, the targeted amino acids are not located within helix 12 (H12 in A) and therefore are unlikely to disturb the structural integrity of the protein. (**C**) Mouse wild-type (WT; upper scheme) and mutant *tuba4a* (three lower schemes) gene locus. Exons (black numbers). Point mutations: E433D, E434D, E441D, E443D, E445D, E447D and E448D. Southern blot probes and expected DNA fragments following *NsiI* or *AflII* digestion (blue or red, respectively). Neo, flanked by frt sites, was removed using FLP-expressing mice. Mutant Tuba4a knock-in mice were generated by replacing wildtype exon 5 with mutant exon 5*. (**D**-**E**) 5´ and 3´ Southern blotting to confirm correct homologous recombination. Wildtype, 12.1 kb. Knock-in, 8.5 kb. (**F**) Long-range PCR. Product of primers I+II: 3.6 kb. (**G**-**H**) 5´ and 3´ Southern blotting following *NsiI* or *AflII*, digestion respectively. (**I**) Representative genotyping PCR. (**J**) Offspring numbers of female and male mice out of 24 litters. (**K**) Genotype distribution for Tuba4aΔpolyGlu (+/+), (+/p) and (p/p) offspring numbers in % shown in (J). (**L**) Data shown in (K) split by sex. The representative micrographs shown in (D-I) are derived from at least three independent experiments. Source data are provided as a Source Data file.

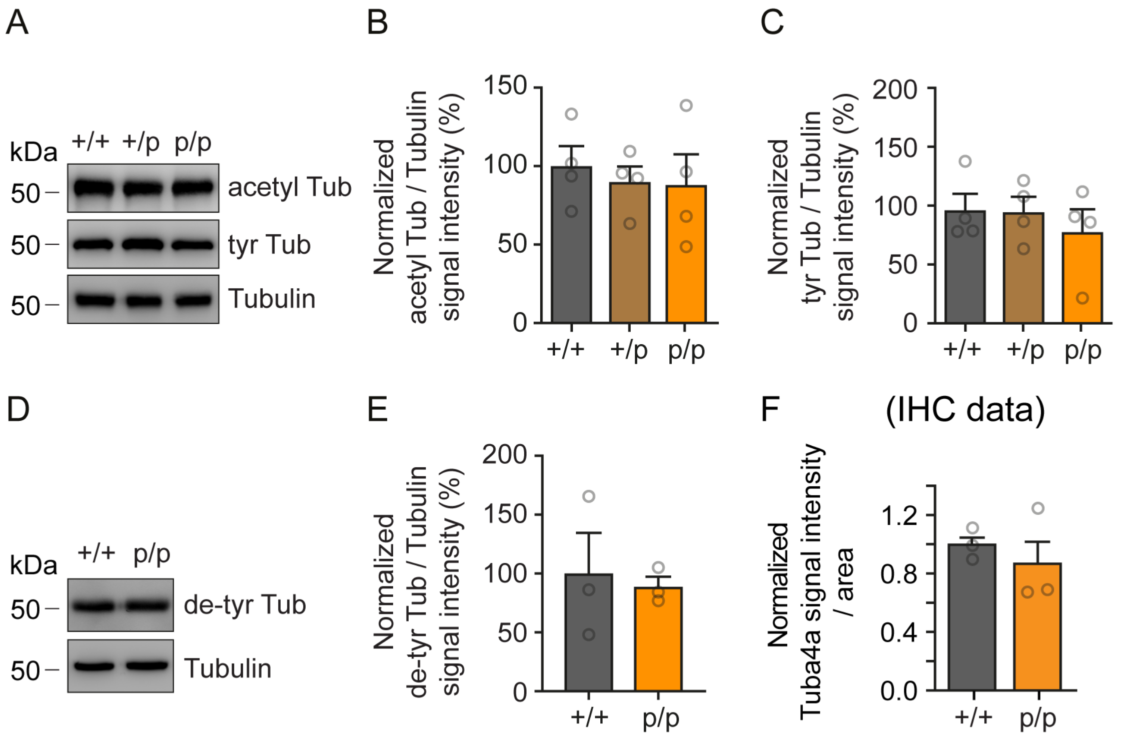


**Supplementary Figure 2, related to Figure 2.** (**A**) Representative western blot analysis depicting acetylated, tyrosinated and total alpha-tubulin protein expression levels in the hippocampus derived from (+/+), (+/p) and (p/p) Tuba4aΔpolyGlu adult mice, as indicated. (**B-C**) Quantification of acetylated and tyrosinated tubulin normalized to alpha-tubulin signal intensities shown in (A). (+/+) set to 100%. n=4 experiments. (**D**) Representative western blot analysis depicting de-tyrosinated and total alpha-tubulin protein expression. (**E**) Quantification of de-tyrosinated tubulin normalized to alpha-tubulin signal intensities shown in (D). (+/+) set to 100%. n=3 experiments. (**F**) Quantification of Tuba4a signal intensities in the CA1 region of the hippocampus normalized to the total area analyzed as shown in Figure 2H. (+/+) set to 1. n=3 experiments. Unpaired Student´s t-test (B, C, E) and Mann-Whitney test (F) were used to asses statistical significance. Data represent as mean ± SEM. Source data, including exact p-values, are provided as a Source Data file.

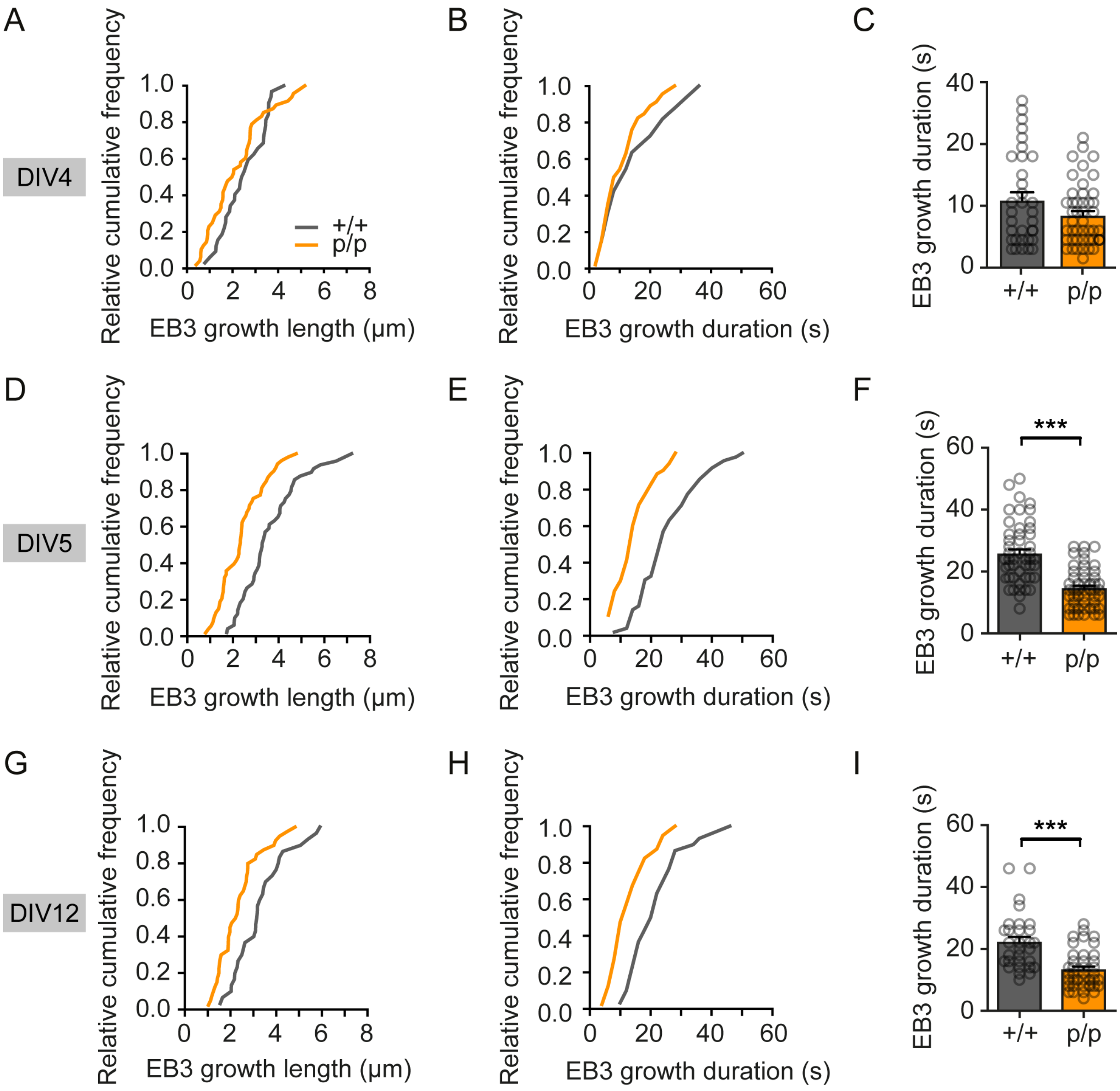


**Supplementary Figure 3, related to Figure 3.** Live imaging data of EB3-GFP in hippocampal neurons at (**A-C**) DIV4, (**D-F**) DIV5 and (**G-I**) DIV12 derived from (+/+) and (p/p) Tuba4aΔpolyGlu mice, as indicated. (A, D, G) Quantification of the relative cumulative frequency of EB3 growth length. (B, E, H) Quantification of the relative cumulative frequency of EB3 growth duration. (C, F, I) Quantification of the mean EB3 growth duration. Mann-Whitney test was used to asses statistical significance. DIV4: n=33(+/+), 46(p/p), DIV5: n=49(+/+), 53(p/p), DIV12: n=30(+/+), 40(p/p) EB3-comets; ***$p<0.001$. Data represent as mean ± SEM. Source data, including exact p-values, are provided as a Source Data file.

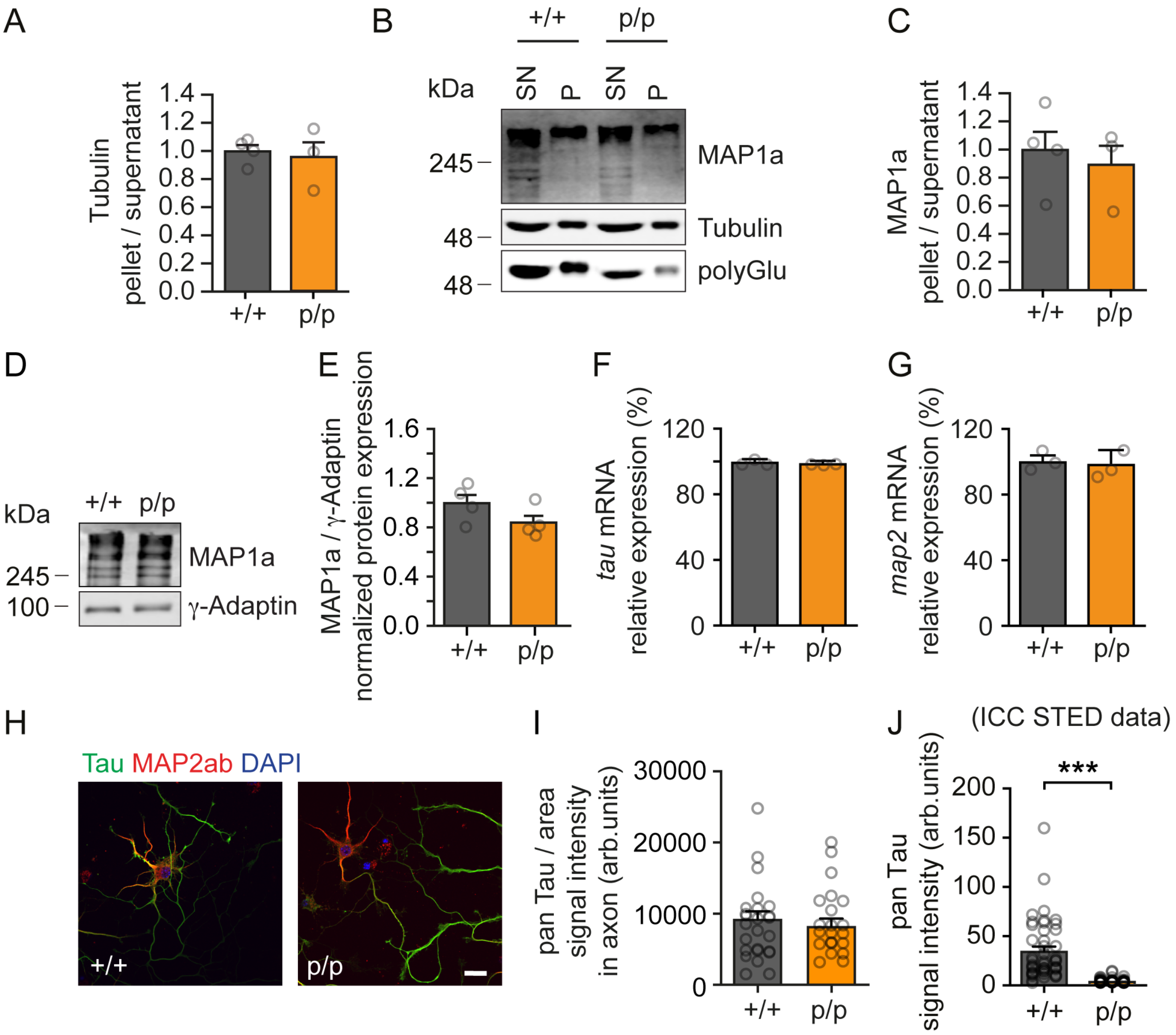


**Supplementary Figure 4, related to Figure 4.** (**A**) Quantification of total alpha Tubulin pellet/supernatant levels using a microtubule pelleting assay, as shown in Figure 4D, indicating comparable amounts of polymerized tubulin (microtubules) between the genotypes. n=4(+/+), 3(p/p) mice per genotype. Unpaired Student's t-test was used to assess statistical significance. (**B-C**) Microtubule pelleting assay after repolymerization of hippocampal tubulin derived from adult (+/+) and (p/p) Tuba4aΔpolyGlu mice, as indicated. (B) Representative western blot analysis depicting MAP1a, total alpha-tubulin and polyglutamylated tubulin. Supernatant (SN): un-polymerized tubulin and dissociated MAPs. Pellet (P): polymerized tubulin (microtubules) and associated MAPs. (C) Quantification of MAP1a pellet/supernatant levels. Ratios smaller 1 indicate higher protein abundance in the supernatant fraction. n=4(+/+), 3(p/p) mice per genotype. (**D**) Representative western blot analysis depicting MAP1a and γ-Adaptin protein expression levels in the hippocampus. (**E**) Quantification of MAP1a normalized to γ-Adaptin

signal intensities shown in (D). (+/+) set to 1. n=4 experiments. (**F**-**G**) Relative *tau* (F) and *map2* (G) mRNA expression levels in the hippocampus. (+/+) set to 100 %. n=3 mice per genotype. (**H**) Co-immunostaining of pan Tau (green) and MAP2a/b (red) using DIV6 hippocampal neurons. DAPI (blue) was used to stain the nuclei. Scale bar, 15 µm. (**I**) Quantification of pan Tau signal intensities shown in H normalized to the area analyzed within 10µm axonal length. n=23 neurons per genotype. (**J**) Quantification of mean pan Tau signal intensities of the super-resolution STED imaging data in axons from DIV14 hippocampal neurons, as shown in Figure 4K. n=45(+/+), 60(p/p) axonal regions per genotype. Arbitrary units (arb. units). Unpaired Student´s t-test (A, C, E-G) and Mann-Whitney test (I, J) were used to asses statistical significance. ***p<0.001. Data represent mean ± SEM. Source data, including exact p-values, are provided as a Source Data file.

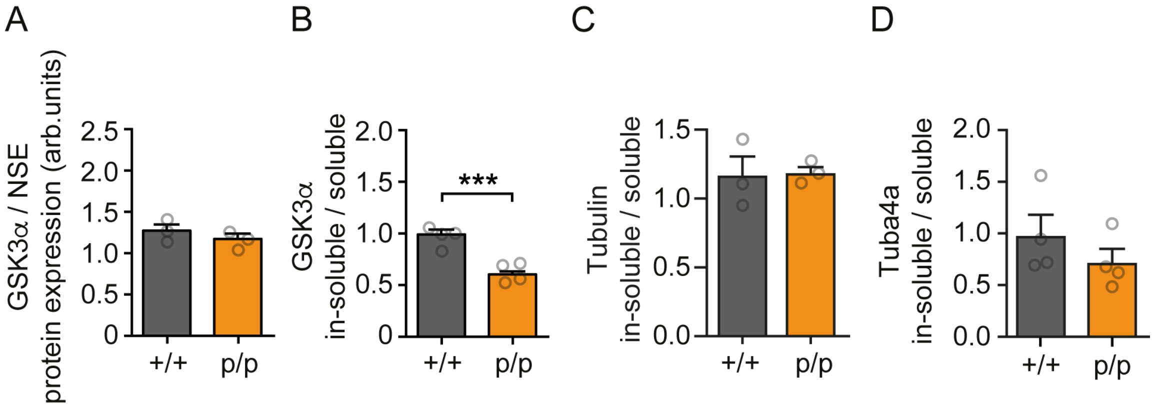


**Supplementary Figure 5, related to Figure 5.** (**A**) Quantification of GSK3α signal intensities determined by western blot analysis of hippocampal lysates derived from adult Tuba4a (+/+) and (p/p) mice, indicating no change in protein expression levels. n=3 mice per genotype. Arbitrary units (arb. units). (**B**-**D**) A soluble-tubulin extraction assay using DIV15 neurons derived from Tuba4aΔpolyGlu (+/+) and (p/p) mice was used to quantify GSK3α (B), total alpha tubulin (C) and Tuba4a (D) in-soluble/soluble levels, as determined by western blot analysis (shown in Figure 5H). Ratios smaller 1 indicate higher protein abundance in the soluble fraction. (B, D) n=4 and (C) n=3 independent cultures per genotype. Unpaired Student´s t-test was used to asses statistical significance. ***p<0.001. Data represent mean ± SEM. Source data, including exact p-values, are provided as a Source Data file.

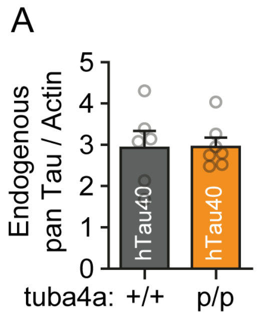


**Supplementary Figure 6, related to Figure 6.** (**A**) Quantification of endogenous pan Tau levels normalized to Actin in adeno virus-transduced CFP-tagged human Tau (hTau40-CFP) DIV19 neurons after 2 days of expression, as shown in Figure 6A (middle WB panel, two left lanes, # endogenous mouse Tau). n=6(+/+), 7(p/p) cultures per genotype. Unpaired Student´s t-test was used to asses statistical significance. Data represent mean ± SEM. Source data, including exact p-values, are provided as a Source Data file.

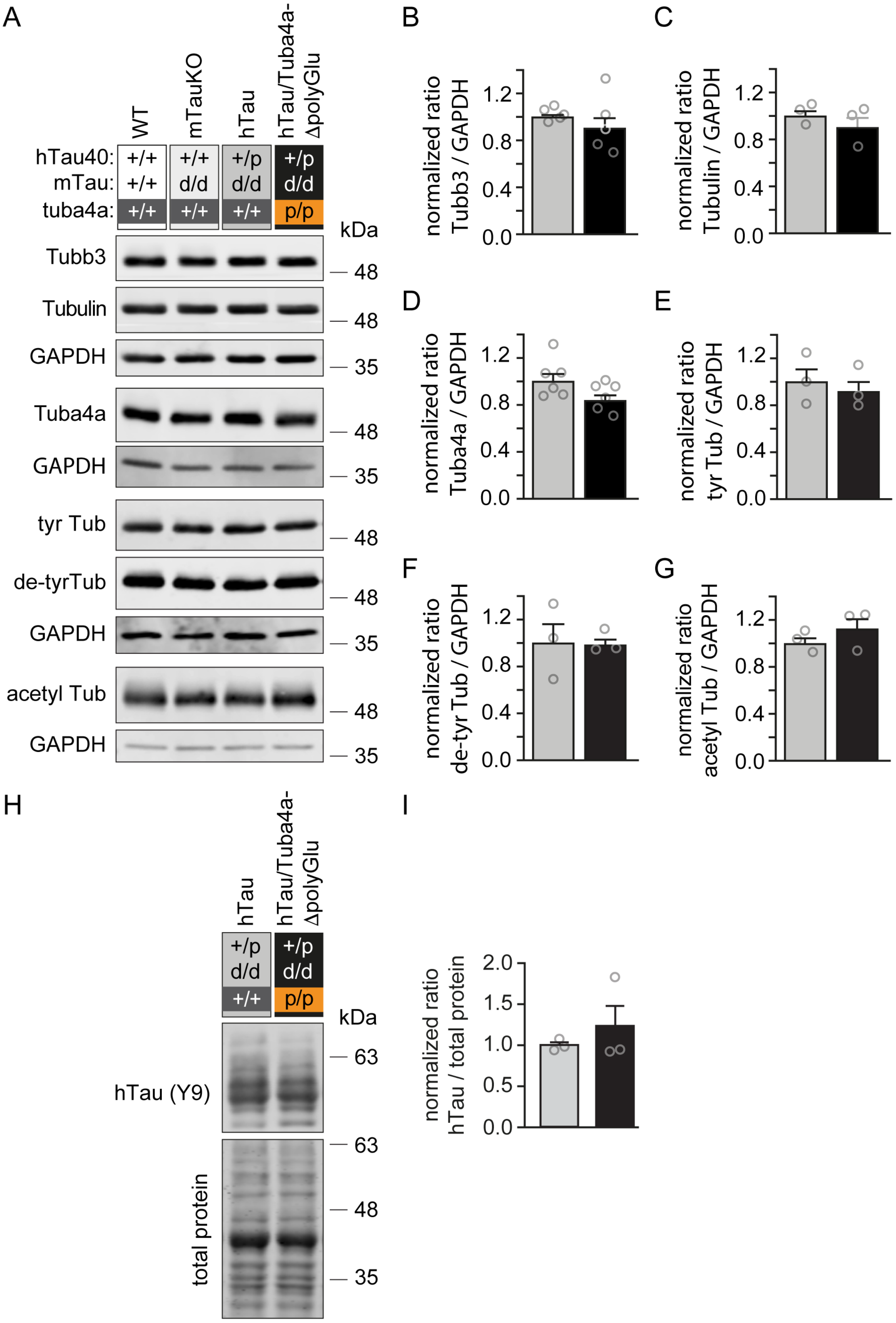


**Supplementary Figure 7, related to Figure 7.** (**A**) Representative western blot analysis depicting Tubb3, total alpha Tubulin, Tuba4a, tyrosinated Tubulin, de-tyrosinated and acetylated Tubulin in cortical extracts derived from 12-month-old (+/+) and (p/p)

Tuba4a∆polyGlu mice, following crossbreeding with hTau (+/p) and mouse(m)Tau knockout (d/d) mice, as indicated. Samples derived from wildtype mice (WT), not expressing hTau, were included as control. GAPDH was used as a loading control. Quantification of (**B**) Tubb3, (**C**) total alpha Tubulin, (**D**) Tuba4a, (**E**) tyrosinated Tubulin, (**F**) de-tyrosinated Tubulin, (**G**) acetylated Tubulin normalized to GAPDH signal intensities as shown in (A). Control (hTau40: +/+, mTau: d/d, tuba4a: +/+) set to 1; (B) n=6, (C) n=3, (D)=6, (E-G) n=3 experiments per genotype. (**H**) Representative western blot analysis depicting hTau (Y9 antibody) in cortical extracts derived from 12-month-old (+/+) and (p/p) Tuba4a∆polyGlu mice, following crossbreeding with hTau (+/p) and mTau (d/d) mice, as indicated. Total protein staining was used as a loading control. (**I**) Quantification of hTau normalized to total protein levels as shown in (H). Control (hTau40: +/+, mTau: d/d, tuba4a: +/+) set to 1, n=3 experiments. Unpaired Student's t-test was used to assess statistical significance. Data represent mean ± SEM. Source data, including exact p-values, are provided as a Source Data file.

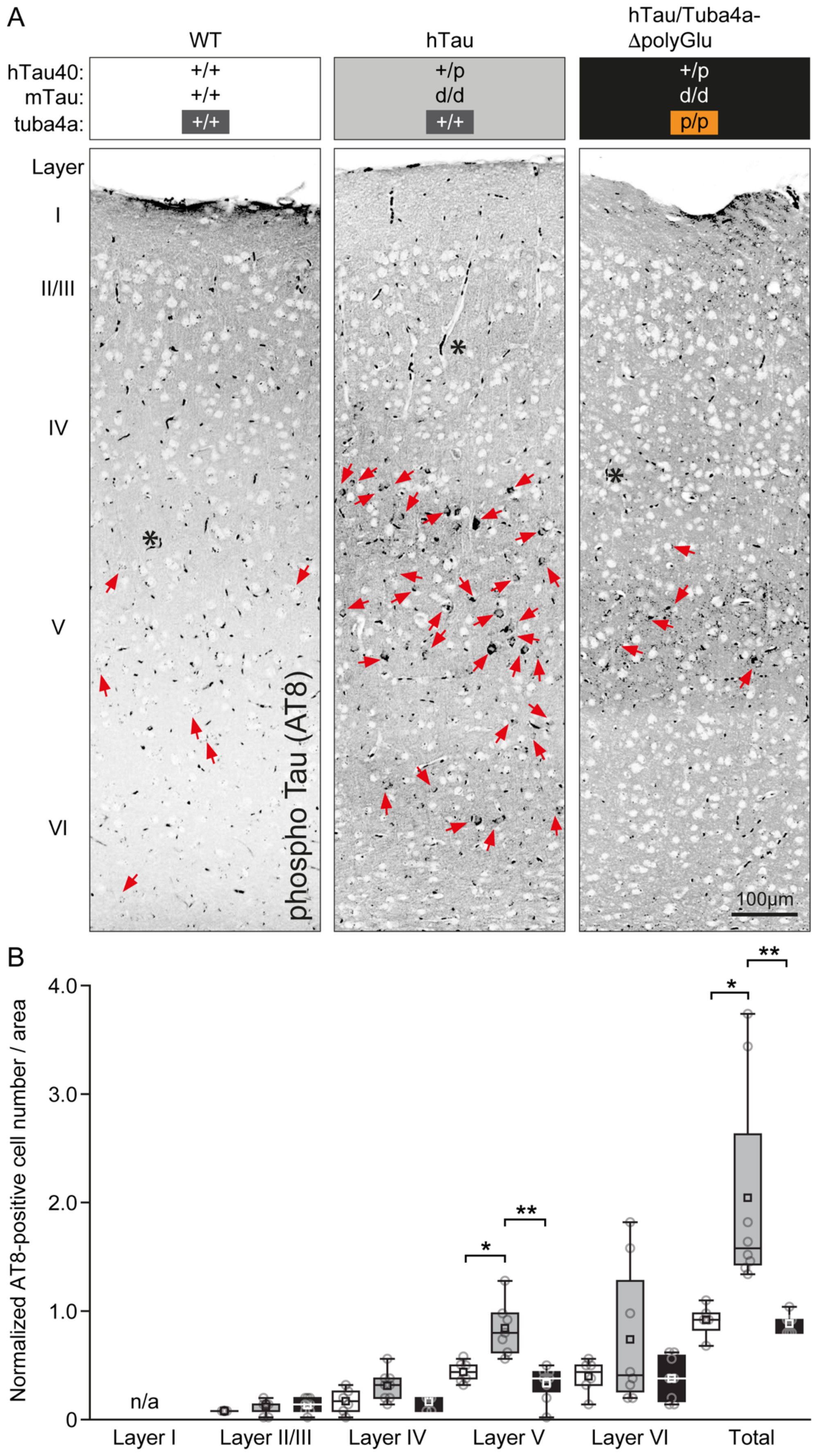
A
WT
hTau
hTau/Tuba4a-ΔpolyGlu
hTau40:
mTau:
tuba4a:
+/+
+/+
+/+
+/p
d/d
+/+
+/p
d/d
p/p
Layer
I
II/III
IV
V
VI
phospho Tau (AT8)
100µm
B
Normalized AT8-positive cell number / area
4.0
3.0
2.0
1.0
0
n/a
Layer I
Layer II/III
Layer IV
Layer V
Layer VI
Total
*
**

**Supplementary Figure 8, related to Figure 7.** (**A**) Immunohistochemical analysis of phosphorylated human Tau in cortical brain sections derived from 12-month-old Tuba4aΔpolyGlu (+/+) and (p/p) mice, following crossbreeding with hTau (+/p) mice. Sections derived from wildtype mice (WT), that did not express hTau, were included as controls. The AT8 antibody (grey signals) was used to detect phosphorylated Tau. Scale bar, 100 µm. Red arrow: AT8-positive cell; Asterisk: Please note that blood vessels are labeled unspecifically (tubular signals). (**B**) Quantification of the relative number of AT8-positive cells per indicated cell layer, normalized to the total area analyzed. WT for total cortex set to 1; n=5(WT), 8(+/+), 7(+/p) per genotype. n/a: not available. Kruskal-Wallis test was used to assess statistical significance. *p<0.05, **p<0.01. Data represented as box plots, median (centre), mean (square), interquartile range (bound of boxes) and minima and maxima (whiskers) are indicated. Source data, including exact p-values, are provided as a Source Data file.

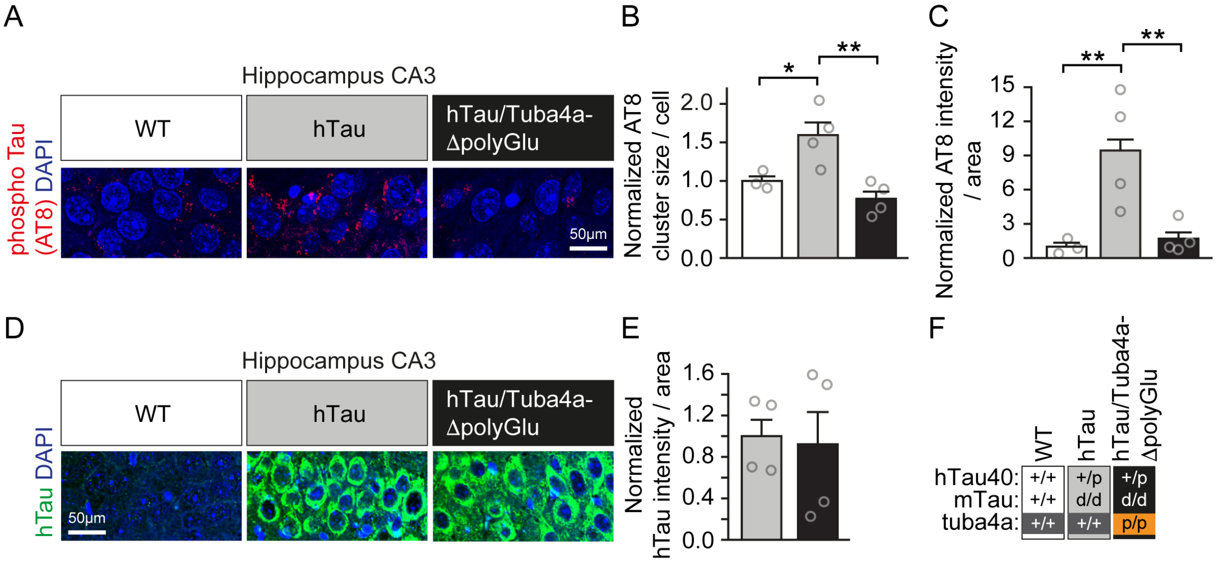


**Supplementary Figure 9, related to Figure 7.** (**A**) Immunohistochemical analysis of subcellular phosphorylated Tau-positive accumulations in CA3 region of hippocampal brain sections derived from 12-month-old Tuba4a (+/+) and (p/p) mice crossed with hTau (+/p) mice. Wildtype sections not expressing hTau, served as controls. The AT8 antibody (red) was used to detect phosphorylated Tau. DAPI (blue) was used to stain the nuclei. Scale bar, 50 µm. Quantification of AT8-positive (**B**) accumulations in size and (**C**) AT8-signal intensities per area

analyzed, shown in (A). WT set to 1; n=3(WT), 4(+/+ and p/p) mice per genotype. Note, Tau phosphorylation is normalized to WT control levels in the genetic background of Tuba4a∆polyGlu. (**D**) Immunohistochemical analysis of total human Tau signal intensities in CA3 region of hippocampal brain sections. The hTau (Y9) antibody (green) was used to detect human Tau. DAPI (blue) was used to stain the nuclei. Scale bar, 50 µm. One-way ANOVA was used to assess statistical significance. (**E**) Quantification of hTau-positive signal intensities normalized to the area analyzed, shown in (D). WT set to 1; n=4 mice per genotype. (**F**) Legend indicating group (condition)/genotype assignment. Unpaired Student's t-test was used to assess statistical significance. *p<0.05, **p<0.01. Data represent mean ± SEM. Source data, including exact p-values, are provided as a Source Data file.

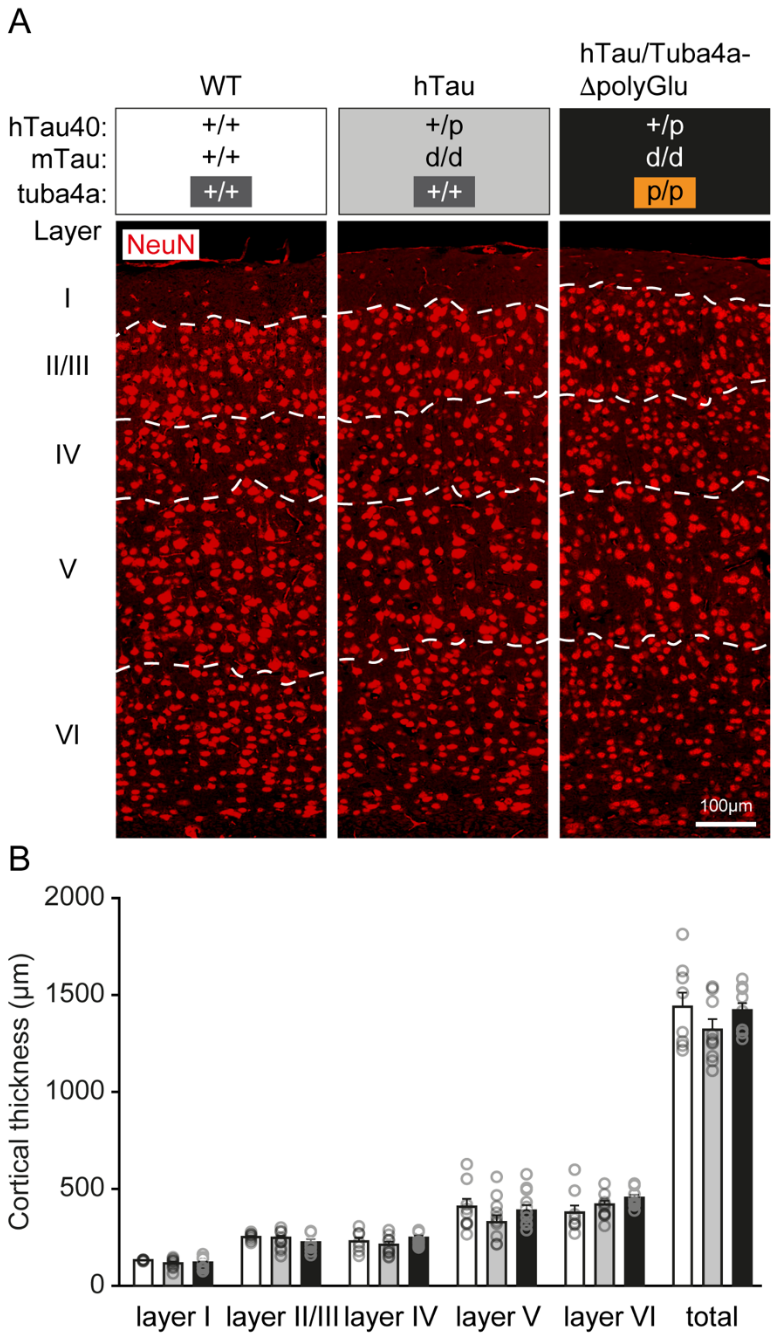


**Supplementary Figure 10, related to Figure 7.** (**A**) Immunohistochemical analysis of total cortical thickness and individual layer thickness based on NeuN immuno-positive signals in cortical brain sections derived from 12-month-old Tuba4aΔpolyGlu (+/+) and (p/p) mice, following crossbreeding with hTau (+/p) mice. Sections derived from wildtype mice (WT), not expressing hTau, were included as control. The NeuN antibody (red signals) was used to detect neuronal cells. Scale bar, 100 µm. (**B**) Quantification of the total cortical thickness and individual layer thickness, as indicated. WT for total cortex thickness set to 1; n=6(WT), 10(+/+ and p/p) per genotype. One-way ANOVA was used to assess statistical significance. Data

represent mean ± SEM. Source data are provided as a Source Data file, including a presentation of exact p-values.

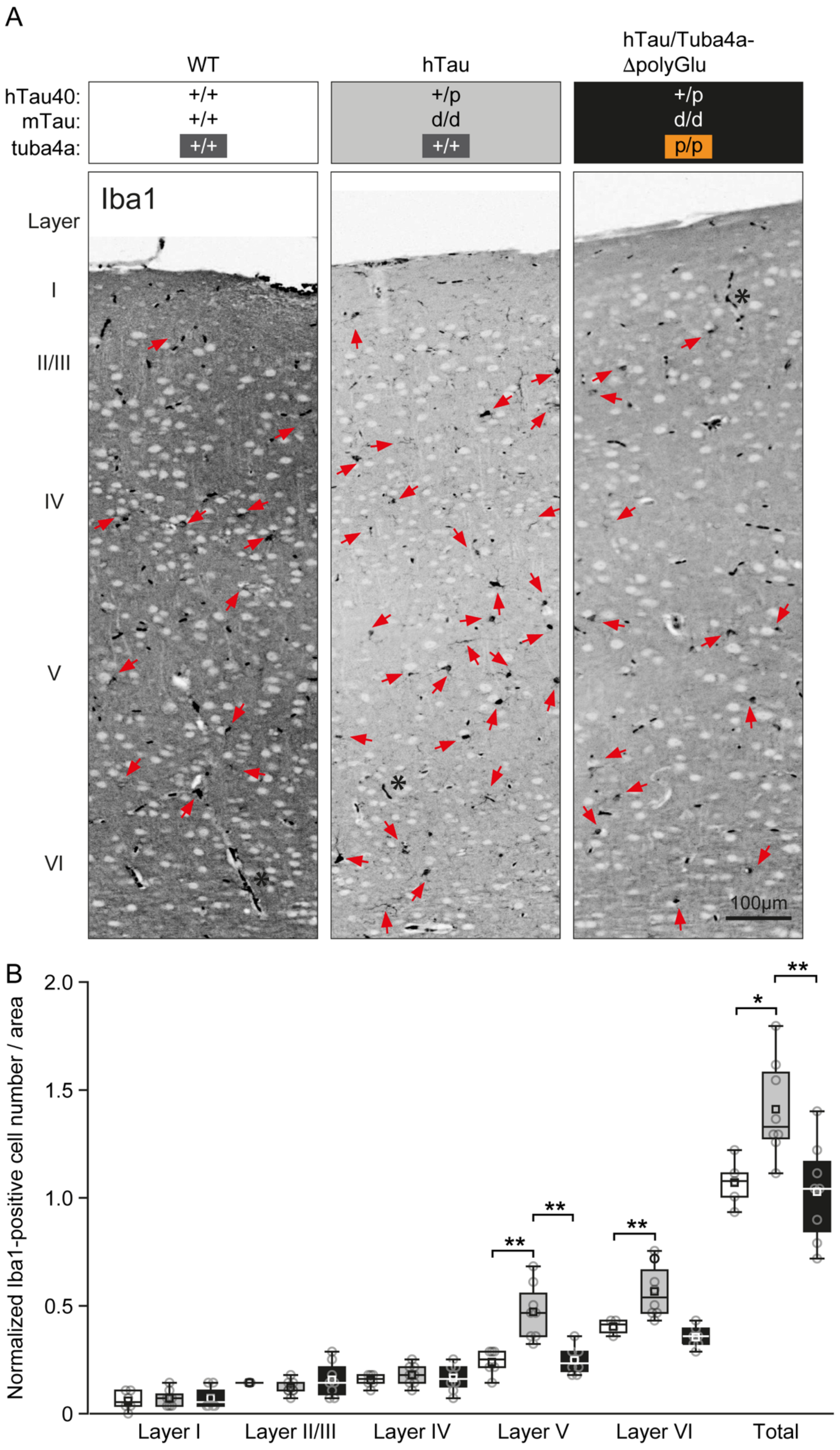
A
WT
hTau
hTau/Tuba4a-ΔpolyGlu
hTau40:
mTau:
tuba4a:
+/+
+/+
+/+
+/p
d/d
+/+
+/p
d/d
p/p
Iba1
Layer
I
II/III
IV
V
VI
100µm
B
Normalized Iba1-positive cell number / area
2.0
1.5
1.0
0.5
0
Layer I
Layer II/III
Layer IV
Layer V
Layer VI
Total
*
**

**Supplementary Figure 11, related to Figure 9.** (**A**) Immunohistochemical analysis of Iba1-positive cells (grey signal), a marker for microglia, in cortical brain sections derived from 12-month-old Tuba4a∆polyGlu (+/+) and (p/p) mice, following crossbreeding with hTau (+/p) mice. Sections derived from wildtype mice (WT), not expressing hTau, were included as control. Scale bar, 100 µm. Red arrow: Iba1-positive cell; Asterisk: Please note that blood vessels are labeled unspecifically (tubular signals). (**B**) Quantification of the relative number of Iba1-positive cells per indicated cell layer, normalized to the total area analyzed. WT for total cortex set to 1; n=4(WT), 8(+/+ and p/p) per genotype. Kruskal-Wallis test was used to assess statistical significance. $^{*}p<0.05$, $^{**}p<0.01$. Data represent as box plots, median (centre), mean (square), inter quartile range (bound of boxes) and minima and maxima (whiskers) are indicated. Source data are provided as a Source Data file, including a presentation of exact p-values.

**Supplementary Tabel 1: Summary of the primary antibodies used in the study.** The name of the antibody, the company, the catalg number, the host species, the clonality and the validation are listed. In addition, the dilution used for western blot (WB), immunohistochemistry (IHC), immunocytochemistry (ICC), STED microscopy (STED) and electron microscopy (EM) are given. The column "Notes" provides further information.

| 1° Antibodies | Company | Catalog # | Species | Clonality | Dilution (WB) | Dilution (IHC) | Dilution (ICC) | STED | EM | Validation | Notes |
|---|---|---|---|---|---|---|---|---|---|---|---|
| anti alpha4a-tubulin | Abcam | AB177479 | rabbit | mono | 1:2000 | 1:1000 | 1:1000 | 1:1000 | 1:500 | Reference: DOI: 10.1002/dneu.22745 | |
| anti-alpha-tubulin | Abcam | AB7291 | mouse | mono | 1:5000 | | 1:1000 | | | Reference: DOI: 10.1002/dneu.22745 | |
| anti beta3-tubulin | Biolegend | 801201 | mouse | mono | 1:5000 | | 1:1000 | | | Reference: DOI: 10.1002/dneu.22745 | |
| anti beta2-tubulin | Abcam | AB151318 | rabbit | poly | 1:1000 | | | | | Reference: DOI: 10.1002/dneu.22745 | unspecifically, AB detects all beta-tubulin subunits |
| anti-polyglutamylated tubulin (GT335) | Adipogen | AG-20B-0020 | mouse | mono | 1:1000 | 1:500 | 1:500 | | | Reference: A. Wolff, et al.; Eur. J. Cell Biol. 59, 425 (1992) | |
| anti-actin | Sigma-Aldrich | A2066 | mouse | mono | 1:5000 | | | | | Antibody Enhanced Validation by Merck | |
| anti-human-pan-Tau | Dako | A0024 | rabbit | poly | 1:2000 | | 1:500 | 1:300 | | KO validated in this study | detects mouse and human |
| anti-human-pan-Tau | Enzo Life Sciences | TA3119 | rabbit | poly | 1:3000 | 1:300 | 1:500 | | | validated in this study by overexpression of hTau | human specific |
| anti-pan-Tau | Synaptic Systems | 314004 | guinea-pig | poly | | | 1:200 | | | Reference: DOI: 10.1038/nprot.2016.111 | detects mouse and human |
| anti-human-PHF-Tau (AT8) | Thermo Fisher Scientific | MN1020 | mouse | mono | 1:1000 | 1:200 | 1:250 | | | antibody verified by cell treatment by manufacturer | detects mouse and human phosphorylated Tau (Ser202/Thr205) |
| anti-human-PHF-Tau (AT270) | Thermo Fisher Scientific | MN1050 | mouse | mono | 1:1000 | | | | | Reference: DOI: 10.1038/s41467-019-11813-6 | detects mouse and human phosphorylated Tau (Thr181) |
| anti-GAPDH | GeneTex | GTX28245 | mouse | mono | 1:5000 | | | | | orthogonal validation by manufacturer | |
| anti-neuronal-specific enolase (NSE) | Novus Biologicals | NB100-1606 | chicken | poly | 1:5000 | | | | | WB detects a single band of the correct moleculare weight | |
| anti-GSK3beta+GSK3alpha | Abcam | EPR18814-102 | rabbit | mono | 1:2000 | | | | | KO validated by manufacturer | |
| anti-Iba1 | Fujifilm Cellular Dynmics | 019-19741 | rabbit | poly | | 1:1000 | | | | Reference: https://doi.org/10.1006/bbrc.2001.5388 | |
| anti-CD68 | Thermo Fisher Scientific | MA5-13324 | mouse | mono | 1:250 | 1:150 | | | | antibody verified by cell treatment by manufacturer | |
| Anti-oligomeric Tau (TOMA-1) | Millipore | MABN819 | mouse | mono | 1:1000 | 1:300 | | | | Reference: DOI: https://doi.org/10.1523/JNEUROSCI.3192-13.2014 | |
| anti-Map1a | Novus Biologicals | NBP2-32630 | rabbit | poly | 1:1000 | | | | | orthogonal validation by manufacturer | |
| anti-Map2a/b (AP-20) | Sigma-Aldrich | M1406 | mouse | mono | 1:1000 | | 1:1000 | | | antibody verified by manufacturer | |
| anti-adaptin Y | BD Transduction Laboratories | 610385 | mouse | mono | 1:1000 | | | | | Reference: DOI: 10.1083/jcb.111.6.2319 | |
| anti-Mark1 | Proteintech | 21552-1-AP | rabbit | poly | 1:1000 | | | | | KD/KO validated by manufacturer | |
| anti-NeuN | Millipore | MAB377 | mouse | mono | | 1:1000 | | | | Reference: DOI: 10.1038/srep14624 | |
| anti-Ankyrin G | Synaptic Systems | 386 004 | guinea-pig | poly | | 1:300 | 1:500 | | | KD validation reference: DOI: 10.1083/jcb.201907048 | |
| anti-Ctip2 | Abcam | ab18465 | rat | mono | | 1:500 | | | | Refernce: DOI: 10.1016/j.neuron.2019.04.013 | |
| anti-acetylated tubulin | Sigma-Aldrich | T7451 | mouse | mono | 1:4000 | | | | | Reference: DOI: 10.1371/journal.pone.0052095 | |
| anti-tyrosine tubulin | Sigma-Aldrich | T 9028 | mouse | mono | 1:1000 | | | | | Reference: DOI: 10.3389/fpls.2015.00937 | |
| anti-de-tyrosinated tubulin | Millipore | AB3201 | rabbit | poly | 1:1000 | | | | | Reference: DOI: 10.1016/j.bpj.2014.05.008 | |

**Supplementary Tabel 2: Summary of the secondary antibodies used in the study.** The name of the antibody, the company, the catalg number and the clonality are listed. In addition, the dilution used for western blot (WB), immunohistochemistry (IHC), immunocytochemistry (ICC), STED microscopy (STED) and electron microscopy (EM) are given.

| 2° Antibodies | Company | Catalog # | Clonality | Dilution (WB) | Dilution (IHC) | Dilution (ICC) | STED | EM |
|---|---|---|---|---|---|---|---|---|
| Peroxidase AffiniPure F(ab')$_2$ Fragment Donkey Anti-Mouse IgG (H+L) | Jackson Immuno Research | 715-036-151 | poly | 1:10000 | | | | |
| Peroxidase AffiniPure Goat Anti-Mouse IgG, light chain specific | Jackson Immuno Research | 115-035-174 | poly | 1:10000 | | | | |
| Peroxidase IgG Fraction Monoclonal Mouse Anti-Rabbit IgG, light chain specific | Jackson Immuno Research | 211-032-171 | poly | 1:10000 | | | | |
| Peroxidase AffiniPure F(ab')$_2$ Fragment Donkey Anti-Rabbit IgG (H+L) | Jackson Immuno Research | 711-036-152 | poly | 1:10000 | | | | |
| Peroxidase AffiniPure Goat Anti-Chicken IgY (IgG) (H+L) | Jackson Immuno Research | 103-035-155 | poly | 1:2500 | | | | |
| IRDye® 680RD Goat Anti-Mouse IgG | LI-COR | 926-68070 | poly | 1:10000 | | | | |
| Alexa Fluor® 790 AffiniPure Goat Anti-Mouse IgG, light chain specific | Jackson Immuno Research | 115-655-174 | poly | 1:5000 | | | | |
| IRDye® 800CW Donkey Anti-Guinea Pig IgG | LI-COR | 926-32411 | poly | 1:10000 | | | | |
| IRDye® 800CW Goat Anti-Rabbit IgG | LI-COR | 926-32211 | poly | 1:10000 | | | | |
| Cy™3 AffiniPure Donkey Anti-Mouse IgG (H+L) | Jackson Immuno Research | 715-165-150 | poly | | 1:500 | 1:500 | | |
| Cy™3 AffiniPure Goat Anti-Mouse IgG, light chain specific | Jackson Immuno Research | 115-165-174 | poly | | 1:500 | 1:500 | | |
| Cy™3 AffiniPure F(ab')$_2$ Fragment Donkey Anti-Rabbit IgG (H+L) | Jackson Immuno Research | 711-166-152 | poly | | 1:500 | 1:500 | | |
| Cy5™-AffiniPure Donkey Anti-Rat IgG (H+L) | Jackson Immuno Research | 712-175-153 | poly | | 1:500 | | | |
| Cy5™-AffiniPure Donkey Anti-Mouse IgG (H+L) | Jackson Immuno Research | 715-175-151 | poly | | 1:500 | | | |
| Cy™5 AffiniPure Donkey Anti-Guinea Pig IgG (H+L) | Jackson Immuno Research | 706-175-148 | poly | | | 1:500 | | |
| Alexa Fluor® 488 AffiniPure F(ab')$_2$ Fragment Donkey Anti-Mouse IgG (H+L) | Jackson Immuno Research | 715-546-150 | poly | | 1:500 | 1:500 | | |
| Alexa Fluor® 488 AffiniPure Donkey Anti-Rabbit IgG (H+L) | Jackson Immuno Research | 711-545-152 | poly | | 1:500 | 1:500 | | |
| Goat Anti-Rabbit IgG STAR RED | Abberior | 2-0012-011-9 | poly | | | | 1:250 | |
| Alexa Fluor® 594 Goat Anti-mouse IgG | Biolegend | 405326 | poly | | | | 1:250 | |
| Goat Anti-rabbit IgG (H+L) 10 nm gold conjugate | Ted Pella Inc. | 17010-1 | poly | | | | | 1:200 |
