# Supplementary material for: Disruption of tubulin-alpha4a polyglutamylation prevents aggregation of hyper-phosphorylated tau and microglia activation in mice
Source: Nat Commun. 2022 Jul 20;13:4192. doi: 10.1038/s41467-022-31776-5 (PMC9300677; doi:10.1038/s41467-022-31776-5)
Supplement: Supplementary file 2 — Reporting Summary [file 41467_2022_31776_MOESM2_ESM.pdf]

nature portfolio

Corresponding author(s): Prof. Dr. Matthias Kneussel
Dr. Torben Johann Hausrat

Last updated by author(s): May 23, 2022

Reporting Summary

Nature Portfolio wishes to improve the reproducibility of the work that we publish. This form provides structure for consistency and transparency in reporting. For further information on Nature Portfolio policies, see our Editorial Policies and the Editorial Policy Checklist.

Statistics

For all statistical analyses, confirm that the following items are present in the figure legend, table legend, main text, or Methods section.

n/a	Confirmed	
☐	☒	The exact sample size (*n*) for each experimental group/condition, given as a discrete number and unit of measurement
☐	☒	A statement on whether measurements were taken from distinct samples or whether the same sample was measured repeatedly
☐	☒	The statistical test(s) used AND whether they are one- or two-sided<br>*Only common tests should be described solely by name; describe more complex techniques in the Methods section.*
☒	☐	A description of all covariates tested
☐	☒	A description of any assumptions or corrections, such as tests of normality and adjustment for multiple comparisons
☐	☒	A full description of the statistical parameters including central tendency (e.g. means) or other basic estimates (e.g. regression coefficient) AND variation (e.g. standard deviation) or associated estimates of uncertainty (e.g. confidence intervals)
☐	☒	For null hypothesis testing, the test statistic (e.g. *F*, *t*, *r*) with confidence intervals, effect sizes, degrees of freedom and *P* value noted<br>*Give P values as exact values whenever suitable.*
☒	☐	For Bayesian analysis, information on the choice of priors and Markov chain Monte Carlo settings
☒	☐	For hierarchical and complex designs, identification of the appropriate level for tests and full reporting of outcomes
☒	☐	Estimates of effect sizes (e.g. Cohen's *d*, Pearson's *r*), indicating how they were calculated

Our web collection on statistics for biologists contains articles on many of the points above.

Software and code

Policy information about availability of computer code

Data collection	Fiji (ImageJ) software version 2.0; Image Studio Lite version 5.2; Meta Morph software version 6.3 r7, ChemoStarTouch version 0.5.77, FluoView-ASW version 2.1b, VisiView 4.0, Abberior Imspector 16.3.13033, Ethovision tracking system (version XT 8.5, Noldus Technology), Relative Expression Software Tool (REST) version REST-2009 (gene-quantification, TU-Munich, Germany).
Data analysis	SPSS 25.0; Prism 5; Meta Morph software version 6.3 r7; Fiji (ImageJ) software version 2.0; Microsoft Excel version 16; Adobe Photoshop version 22.3.0, Ethovision tracking system (version XT 8.5, Noldus Technology), Relative Expression Software Tool (REST) version REST-2009 (gene-quantification, TU-Munich, Germany).

For manuscripts utilizing custom algorithms or software that are central to the research but not yet described in published literature, software must be made available to editors and reviewers. We strongly encourage code deposition in a community repository (e.g. GitHub). See the Nature Portfolio guidelines for submitting code & software for further information.

Data

Policy information about availability of data

All manuscripts must include a data availability statement. This statement should provide the following information, where applicable:

- Accession codes, unique identifiers, or web links for publicly available datasets
- A description of any restrictions on data availability
- For clinical datasets or third party data, please ensure that the statement adheres to our policy

Source data are provided with this paper. Individual data points underlying all reported means/averages in box plots, bar charts and line graphs are provided in

separate Excel spread sheets labeled according to the figures and sub-figures of the main manuscript and the supplemental information. In addition, uncropped versions of gels or blots, labeled with the relevant information, are included.

Human research participants

Policy information about studies involving human research participants and Sex and Gender in Research.

Reporting on sex and gender	*Use the terms sex (biological attribute) and gender (shaped by social and cultural circumstances) carefully in order to avoid confusing both terms. Indicate if findings apply to only one sex or gender; describe whether sex and gender were considered in study design whether sex and/or gender was determined based on self-reporting or assigned and methods used. Provide in the source data disaggregated sex and gender data where this information has been collected, and consent has been obtained for sharing of individual-level data; provide overall numbers in this Reporting Summary. Please state if this information has not been collected. Report sex- and gender-based analyses where performed, justify reasons for lack of sex- and gender-based analysis.*
Population characteristics	*Describe the covariate-relevant population characteristics of the human research participants (e.g. age, genotypic information, past and current diagnosis and treatment categories). If you filled out the behavioural & social sciences study design questions and have nothing to add here, write "See above."*
Recruitment	*Describe how participants were recruited. Outline any potential self-selection bias or other biases that may be present and how these are likely to impact results.*
Ethics oversight	*Identify the organization(s) that approved the study protocol.*

Note that full information on the approval of the study protocol must also be provided in the manuscript.

Field-specific reporting

Please select the one below that is the best fit for your research. If you are not sure, read the appropriate sections before making your selection.

[x] Life sciences [ ] Behavioural & social sciences [ ] Ecological, evolutionary & environmental sciences

For a reference copy of the document with all sections, see nature.com/documents/nr-reporting-summary-flat.pdf

Life sciences study design

All studies must disclose on these points even when the disclosure is negative.

Sample size	Sample size was determined based on pilot experiments and/or a g-power calculation using G*Power 3 version 3.1.3.
Data exclusions	Including outliers in the statistical analysis would lead to false positive or false negative results. Therefore, in the following experiments, outliers (based on "data exploration" using SPSS) were excluded from the analysis:<br><br>Figure7B: 1 value removed for p/p<br>Figure7C: 3 values removed for +/+<br>Figure8G: 1 value removed for p/p<br>Figure8H: 1 value removed for p/p<br>Suppl.Figure8B: Layer II/III 2 values removed for WT; Layer IV 1 value removed for p/p; Layer V 1 value removed for +/+; total 1 value removed for p/p<br>Suppl.Figure 9B: 1 value removed for p/p<br>Suppl.Figure 9C: 1 value removed for p/p<br>Suppl.Figure10B: Layer I 2 values removed for WT; Layer II/III 1 value removed for p/p; Layer IV 1 value removed for WT; Layer VI 1 value removed for p/p<br>Suppl.Figure11B: Layer II/III 2 values removed for WT; Layer VI 2 values removed for WT 1 value removed for p/p; total 1 value removed for WT
Replication	All attempts at replication were successful.
Randomization	The investigators randomly allocated the samples to the different experimental groups.
Blinding	The investigators were not blinded to group allocation during data collections and analysis due to the experimental design.

Reporting for specific materials, systems and methods

We require information from authors about some types of materials, experimental systems and methods used in many studies. Here, indicate whether each material, system or method listed is relevant to your study. If you are not sure if a list item applies to your research, read the appropriate section before selecting a response.

Materials & experimental systems

n/a	Involved in the study
☐	☒ Antibodies
☐	☒ Eukaryotic cell lines
☒	☐ Palaeontology and archaeology
☐	☒ Animals and other organisms
☒	☐ Clinical data
☒	☐ Dual use research of concern

Methods

n/a	Involved in the study
☒	☐ ChIP-seq
☒	☐ Flow cytometry
☒	☐ MRI-based neuroimaging

Antibodies

Antibodies used

1° Antibodies
Company
Catalog #
Validation

anti alpha4a-tubulin
Abcam
AB177479
Reference: DOI: 10.1002/dneu.22745

anti-alpha-tubulin
Abcam
AB7291
Reference: DOI: 10.1002/dneu.22745

anti beta3-tubulin
Biolegend
801201
Reference: DOI: 10.1002/dneu.22745

anti beta2-tubulin
Abcam
AB151318
Reference: DOI: 10.1002/dneu.22745

anti-polyglutamylated tubulin (GT335)
Adipogen
AG-20B-0020
Reference: A. Wolff, et al.; Eur. J. Cell Biol. 59, 425 (1992)

anti-actin
Sigma-Aldrich
A2066
Antibody Enhanced Validation by Merck

anti-human-pan-Tau
Dako
A0024
KO validated in this study

anti-human-pan-Tau
Enzo Life Sciences
TA3119
validated in this study by overexpression of hTau

anti-pan-Tau
Synaptic Systems
314004
Reference: DOI: 10.1038/nprot.2016.111

anti-human-PHF-Tau (AT8)

Thermo Fisher Scientific
MN1020
antibody verified by cell treatment by manufacturer

anti-human-PHF-Tau (AT270)
Thermo Fisher Scientific
MN1050
Reference: DOI: 10.1038/s41467-019-11813-6

anti-GAPDH
GeneTex
GTX28245
orthogonal validation by manufacturer

anti-neuronal-specific enolase (NSE)
Novus Biologicals
NB100-1606
WB detects a single band of the correct moleculare weight

anti-GSK3beta+GSK3alpha
Abcam
EPR18814-102
KO validated by manufacturer

anti-Iba1
Fujifilm Cellular Dynmics
019-19741
Reference: https://doi.org/10.1006/bbrc.2001.5388

anti-CD68
Thermo Fisher Scientific
MA5-13324
antibody verified by cell treatment by manufacturer

Anti-oligomeric Tau (TOMA-1)
Millipore
MABN819
Reference: DOI: https://doi.org/10.1523/JNEUROSCI.3192-13.2014

anti-Map1a
Novus Biologicals
NBP2-32630
orthogonal validation by manufacturer

anti-Map2a/b (AP-20)
Sigma-Aldrich
M1406
antibody verified by manufacturer

anti-adaptin Y
BD Transduction Laboratories
610385
Reference: DOI: 10.1083/jcb.111.6.2319

anti-Mark1
Proteintech
21552-1-AP
KD/KO validated by manufacturer

anti-NeuN
Millipore
MAB377
Reference: DOI: 10.1038/srep14624

anti-Ankyrin G
Synaptic Systems
386 004
KD validation reference: DOI: 10.1083/jcb.201907048

anti-Ctip2
Abcam
ab18465
Refernce: DOI: 10.1016/j.neuron.2019.04.013

anti-acetylated tubulin
Sigma-Aldrich
T7451
Reference: DOI: 10.1371/journal.pone.0052095

anti-tyrosine tubulin
Sigma-Aldrich
T 9028
Reference: DOI: 10.3389/fpls.2015.00937

anti-de-tyrosinated tubulin
Millipore
AB3201
Reference: DOI: 10.1016/j.bpj.2014.05.008

Validation

1° Antibodies
Company
Catalog #
Validation

anti alpha4a-tubulin
Abcam
AB177479
Reference: DOI: 10.1002/dneu.22745

anti-alpha-tubulin
Abcam
AB7291
Reference: DOI: 10.1002/dneu.22745

anti beta3-tubulin
Biolegend
801201
Reference: DOI: 10.1002/dneu.22745

anti beta2-tubulin
Abcam
AB151318
Reference: DOI: 10.1002/dneu.22745

anti-polyglutamylated tubulin (GT335)
Adipogen
AG-20B-0020
Reference: A. Wolff, et al.; Eur. J. Cell Biol. 59, 425 (1992)

anti-actin
Sigma-Aldrich
A2066
Antibody Enhanced Validation by Merck

anti-human-pan-Tau
Dako
A0024
KO validated in this study

anti-human-pan-Tau
Enzo Life Sciences
TA3119
validated in this study by overexpression of hTau

anti-pan-Tau
Synaptic Systems
314004

Reference: DOI: 10.1038/nprot.2016.111

anti-human-PHF-Tau (AT8)
Thermo Fisher Scientific
MN1020
antibody verified by cell treatment by manufacturer

anti-human-PHF-Tau (AT270)
Thermo Fisher Scientific
MN1050
Reference: DOI: 10.1038/s41467-019-11813-6

anti-GAPDH
GeneTex
GTX28245
orthogonal validation by manufacturer

anti-neuronal-specific enolase (NSE)
Novus Biologicals
NB100-1606
WB detects a single band of the correct moleculare weight

anti-GSK3beta+GSK3alpha
Abcam
EPR18814-102
KO validated by manufacturer

anti-Iba1
Fujifilm Cellular Dynmics
019-19741
Reference: https://doi.org/10.1006/bbrc.2001.5388

anti-CD68
Thermo Fisher Scientific
MA5-13324
antibody verified by cell treatment by manufacturer

Anti-oligomeric Tau (TOMA-1)
Millipore
MABN819
Reference: DOI: https://doi.org/10.1523/JNEUROSCI.3192-13.2014

anti-Map1a
Novus Biologicals
NBP2-32630
orthogonal validation by manufacturer

anti-Map2a/b (AP-20)
Sigma-Aldrich
M1406
antibody verified by manufacturer

anti-adaptin Y
BD Transduction Laboratories
610385
Reference: DOI: 10.1083/jcb.111.6.2319

anti-Mark1
Proteintech
21552-1-AP
KD/KO validated by manufacturer

anti-NeuN
Millipore
MAB377
Reference: DOI: 10.1038/srep14624

anti-Ankyrin G

Synaptic Systems
386 004
KD validation reference: DOI: 10.1083/jcb.201907048

anti-Ctip2
Abcam
ab18465
Refernce: DOI: 10.1016/j.neuron.2019.04.013

anti-acetylated tubulin
Sigma-Aldrich
T7451
Reference: DOI: 10.1371/journal.pone.0052095

anti-tyrosine tubulin
Sigma-Aldrich
T 9028
Reference: DOI: 10.3389/fpls.2015.00937

anti-de-tyrosinated tubulin
Millipore
AB3201
Reference: DOI: 10.1016/j.bpj.2014.05.008

Eukaryotic cell lines

Policy information about cell lines and Sex and Gender in Research

Cell line source(s)	C57BL/6 mice; male and female; embryonic day 16
Authentication	Primary hippocampal neurons.
Mycoplasma contamination	Cells were not tested for mycoplasma contamination, because they were isolated directly from living tissue.
Commonly misidentified lines (See ICLAC register)	No misidentified cell lines were used in the study.

Animals and other research organisms

Policy information about studies involving animals; ARRIVE guidelines recommended for reporting animal research, and Sex and Gender in Research

Laboratory animals	Tuba4a"delta"polyGlu mice, C57BL/6, male and female, 3-month- and 12-month-old; FLP-Deleter mice, C57BL/6, male and female, 3-month-old; CMV-Cre mice, C57BL/6, male and female, 3-month-old.
Wild animals	No.
Reporting on sex	Always, male and female mice were used. This is stated in the method section.
Field-collected samples	No.
Ethics oversight	All animal experiments complied with all ethical regulations for animal testing and research in accordance with the European Communities Council Directive (2010/63/EU) and were approved by the ethics committees of the city-state of Hamburg (Behörde für Justiz und Verbraucherschutz, Fachbereich Lebensmittelsicherheit und Veterinärwesen (reference (ID number) 100/13) and the animal care committee of the University Medical Center Hamburg-Eppendorf.

Note that full information on the approval of the study protocol must also be provided in the manuscript.
